# New corticioid taxa in Phanerochaetaceae (Polyporales, Basidiomycota) from East Asia

**DOI:** 10.3389/fmicb.2023.1093096

**Published:** 2023-03-09

**Authors:** Yue Li, Che-Chih Chen, Shuang-Hui He

**Affiliations:** ^1^School of Ecology and Nature Conservation, Beijing Forestry University, Beijing, China; ^2^Department of Biology, National Museum of Natural Science, Taichung, Taiwan

**Keywords:** phlebioid clade, phylogeny, taxonomy, white rot, wood-decaying fungi

## Abstract

The species diversity, taxonomy, and phylogeny of five corticioid genera of Phanerochaetaceae, namely, *Hyphodermella, Roseograndinia, Phlebiopsis, Rhizochaete*, and *Phanerochaete*, in East Asia are studied by using the morphological and molecular methods. Phylogenetic analyses were performed separately for the *Donkia, Phlebiopsis, Rhizochaete*, and *Phanerochaete* clades based on ITS1-5.8S-ITS2 and nrLSU sequence data. In total, seven new species were found, two new combinations are suggested, and a new name is proposed. In the *Donkia* clade, *Hyphodermella* sensu stricto was strongly supported with two new lineages, namely *H. laevigata* and *H. tropica*, which were recovered. *Hyphodermella aurantiaca* and *H. zixishanensis* are members of *Roseograndinia*, while *R. jilinensis* is proved to be a later synonym of *H. aurantiaca*. In the *Phlebiopsis* clade, *P. cana* sp. nov. was found on the bamboo from tropical Asia. In the *Rhizochaete* clade, four new species, *R. nakasoneae, R. subradicata, R. terrestris*, and *R. yunnanensis* were recovered based mainly on molecular analyses. In the *Phanerochaete* clade, *P. subsanguinea* nom. nov. is proposed to replace *Phanerochaete rhizomorpha* C.L. Zhao & D.Q. Wang, which is an invalid name because it was published after *Phanerochaete rhizomorpha* C.C. Chen, Sheng H. Wu & S.H. He, representing another species. Descriptions and illustrations are provided for the new species, and discussions are given for new taxa and names. Identification keys to *Hyphodermella* species worldwide and *Rhizochaete* species in China are given separately.

## Introduction

The species diversity, taxonomy, and phylogeny of the large phlebioid clade, which includes three families, namely, Phanerochaetaceae, Irpicaceae and Meruliaceae, have been intensively studied in recent years, and the number of taxa has been dramatically increased (Floudas and Hibbett, 2015; Miettinen et al., 2016; Justo et al., 2017; Nakasone et al., 2017, 2021; Chen et al., 2018b, 2020, 2021, 2022; Ma and Zhao, 2019; Huang and Zhao, 2020; Xu Y. L. et al., 2020; Gu and Zhao, 2021; Zhao et al., 2021; Li et al., 2022). To date, 60 genera of poroid, corticioid, and hydnoid fungi are recognized in the three families, which mostly cause white rot on both angiosperms and gymnosperms (Chen et al., 2021; Nakasone et al., 2021; Lira et al., 2022). Among corticioid fungi, *Phanerochaete* s.l. and *Phlebia* s.l. are the two largest groups in the clade, which have been divided into many small genera with the aid of molecular systematics.

East Asia, especially the tropic areas, has been shown to be rich in the species diversity of the corticioid fungi of the phlebioid clade (Ma and Zhao, 2019; Huang and Zhao, 2020; Xu Y. L. et al., 2020; Chen et al., 2021; Gu and Zhao, 2021; Li et al., 2021, 2022; Zhao et al., 2021). Although many new taxa were described from this region, the species diversity, especially for the cryptic species, has not been completely understood, and the generic positions of some species need to be further studied (Chen et al., 2021). In this study, we carried out complete taxonomic and phylogenetic studies on the newly collected specimens and some newly described taxa of corticioid fungi in Phanerochaetaceae from East Asia. Seven new species in *Hyphodermella, Phlebiopsis*, and *Rhizochaete* were found, and two new combinations of *Roseograndinia* and a new name for *Phanerochaete* are proposed. These results are continuations and supplements to the research of phlebioid fungi in China.

## Materials and methods

### Specimen collection

Field trips for specimen collection in several nature reserves and forest parks in China and other countries were carried out by the authors. *In situ* photos of the fungi were taken with a Canon EOS 70D camera (Canon Corporation, Japan). Fresh specimens were dried at 40°C with a portable drier (manufactured in Finland). Dried specimens were labeled and then stored in a refrigerator at −40°C for 2 weeks to kill the insects and their eggs before they were ready for morphological and molecular studies. Voucher specimens are deposited at the herbaria of Beijing Forestry University, Beijing, China (BJFC), the National Museum of Natural Science, Taichung, Taiwan (TNM), and the Center for Forest Mycology Research, Madison, Wisconsin, U.S.A. (CFMR). Herbarium code designations follow the Index Herbariorum.^1^

### Morphological studies

Thin, freehand sections were made from dried basidiomes and mounted in 2% (w/v) aqueous potassium hydroxide (KOH) and 1% (w/v) aqueous phloxine. Amyloidity and dextrinoidity of basidiospores were checked in Melzer's reagent (IKI). Cyanophily of hyphal and basidiospore walls was observed in 1% (weight/volume) cotton blue in 60% (w/v) lactic acid (CB). Microscopic examinations were carried out with a Nikon Eclipse 80i microscope (Nikon Corporation, Japan) at magnifications up to 1,000×. Drawings were made with the aid of a drawing tube. The following abbreviations are used: IKI– = neither amyloid nor dextrinoid, CB– = acyanophilous, L = mean spore length, W = mean spore width, Q = L/W ratio, and n (a/b) = the number of spores (a) measured from the number of specimens (b). Color codes and names were followed as suggested by Kornerup and Wanscher (1978).

### DNA extraction and sequencing

A CTAB plant genomic DNA extraction kit, DN14 (Aidlab Biotechnologies Co., Ltd, Beijing, China), was used to extract total genomic DNA from dried specimens, which was then amplified by the polymerase chain reaction (PCR), according to the manufacturer's instructions. The ITS1-5.8S-ITS2 region (ITS) was amplified with the primer pair ITS5/ITS4 (White et al., 1990) using the following protocol: initial denaturation at 95°C for 4 min, followed by 34 cycles at 94°C for 40 s, 58°C for 45 s, 72°C for 1 min, and the final extension at 72°C for 10 min. The nrLSU D1-D2 region (nrLSU) was amplified with the primer pair LR0R/LR7^2^ using the following procedure: initial denaturation at 94°C for 1 min, followed by 34 cycles at 94°C for 30 s, 50°C for 1 min, 72°C for 1.5 min, and the final extension at 72°C for 10 min. DNA sequencing was performed at the Beijing Genomics Institute, and the sequences were deposited in GenBank^3^ (Table 1). BioEdit v.7.0.5.3 (Hall, 1999) and Geneious Basic v.11.1.15 (Kearse et al., 2012) were used to review the chromatograms and for contig assembly.

**Table 1:** Species and sequences used in the phylogenetic analyses.

**Taxa**	**Voucher**	**Locality**	**ITS**	**nrLSU**	**References**
*Alboefibula bambusicola*	Chen 2304	China	MZ636926	MZ637091	Chen et al., 2021
*Alboefibula bambusicola*	Wu 1209-26	China	MZ636927	MZ637092	Chen et al., 2021
*Alboefibula gracilis*	Wu 0606-83	China	MZ636928	MZ637093	Chen et al., 2021
*Alboefibula gracilis*	Wu 1809-106	China	MZ636929	MZ637094	Chen et al., 2021
*Crepatura ellipsospora*	CLZhao 697	China	MK343695	MK343699	Ma and Zhao, 2019
*Crepatura ellipsospora*	CLZhao 1265	China	MK343692	MK343696	Ma and Zhao, 2019
*Donkia pulcherrima*	GC 1707-11	China	LC378994	LC379152	Chen et al., 2018b
*Donkia pulcherrima*	Gothenburg-2022	Austria	KX752591	KX752591	Miettinen et al., 2016
*Efibulella deflectens*	FCUG 1568	Sweden	AF141619	AF141619	Parmasto and Hallenberg, 2000
*Geliporus exilisporus*	Dai 2172	China	KU598211	KU598216	Yuan et al., 2017
*Geliporus exilisporus*	GC 1702-15	China	LC378995	LC379153	Chen et al., 2018b
*Hyphodermella corrugata*	MA-Fungi 5527	Morocco	FN600372	JN939597	Telleria et al., 2010
*Hyphodermella corrugata*	MA-Fungi 24238	Portugal	FN600378	JN939586	Telleria et al., 2010
***Hyphodermella laevigata***	**He 5427** ^*^	**China**	**ON964013**	**ON963996**	**Present study**
***Hyphodermella laevigata***	**He 5430**	**China**	**ON964014**	**ON963997**	**Present study**
*Hyphodermella pallidostraminea*	LE 286968	Russia	OK138912	OK138911	Unpublished
“*Hyphodermella” poroides*	Dai 10848	China	KX008368	KX011853	Zhao et al., 2017
“*Hyphodermella” poroides*	Dai 12045	China	KX008367	KX011852	Zhao et al., 2017
*Hyphodermella rosae*	FP-150552	USA	KP134978	KP135223	Floudas and Hibbett, 2015
*Hyphodermella rosae*	GC 1604-113	China	MZ636986	MZ637147	Chen et al., 2021
***Hyphodermella tropica***	**He 3808**	**China**	**ON964010**	**ON963993**	**Present study**
***Hyphodermella tropica***	**He 3993** ^*^	**China**	**ON964011**	**ON963994**	**Present study**
***Hyphodermella tropica***	**He 4004**	**China**	**ON964012**	**ON963995**	**Present study**
*Odontoefibula orientalis*	GC 1703-76	China	LC379004	LC379156	Chen et al., 2018b
*Odontoefibula orientalis*	Wu 0910-57	China	LC363490	LC363495	Chen et al., 2018b
*Phanerochaete albida*	GC 1407-14	China	MZ422788	MZ637179	Floudas and Hibbett, 2015
*Phanerochaete albida*	WEI 18-365	China	MZ422789	MZ637180	Floudas and Hibbett, 2015
*Phanerochaete alnea ssp. alnea*	FP-151125	USA	KP135177	MZ637181	Floudas and Hibbett, 2015; Chen et al., 2021
*Phanerochaete alnea ssp. lubrica*	HHB-13753	USA	KP135178	—	Floudas and Hibbett, 2015
*Phanerochaete arizonica*	RLG-10248-Sp	USA	KP135170	KP135239	Floudas and Hibbett, 2015
*Phanerochaete australosanguinea*	MA-Fungi 91308	Chile	MH233925	MH233928	Phookamsak et al., 2019
*Phanerochaete australosanguinea*	MA-Fungi 91309	Chile	MH233926	MH233929	Phookamsak et al., 2019
*Phanerochaete burtii*	FD-171	USA	KP135116	—	Floudas and Hibbett, 2015
*Phanerochaete burtii*	HHB-4618-Sp	USA	KP135117	KP135241	Floudas and Hibbett, 2015
*Phanerochaete calotricha*	Vanhanen-382	Finland	KP135107	—	Floudas and Hibbett, 2015
*Phanerochaete canolutea*	Wu 9211-105	China	MZ422795	GQ470641	Wu et al., 2010; Chen et al., 2021
*Phanerochaete canolutea*	Wu 9712-18	China	MZ422796	GQ470656	Wu et al., 2010; Chen et al., 2021
*Phanerochaete carnosa*	FD-474	USA	KP135126	—	Floudas and Hibbett, 2015
*Phanerochaete carnosa*	HHB-9195-Sp	USA	KP135129	KP135242	Floudas and Hibbett, 2015
*Phanerochaete citrinosanguinea*	FD-287	USA	KP135095	—	Floudas and Hibbett, 2015
*Phanerochaete citrinosanguinea*	FP-105385	USA	KP135100	KP135234	Floudas and Hibbett, 2015
*Phanerochaete hainanensis*	He 3562	China	MT235692	MT248179	Boonmee et al., 2021
*Phanerochaete leptocystidiata*	Dai 10468	China	MT235684	MT248167	Xu Y. L. et al., 2020
*Phanerochaete leptocystidiata*	He 5853	China	MT235685	MT248168	Xu Y. L. et al., 2020
*Phanerochaete pseudosanguinea*	FD-244	USA	KP135098	KP135251	Floudas and Hibbett, 2015
*Phanerochaete rhizomorpha*	GC 1708-335	China	MZ422824	MZ637208	Chen et al., 2021
*Phanerochaete rhizomorpha*	Wu 0910-61	China	MZ422826	MZ637210	Chen et al., 2021
*Phanerochaete rhodella*	FD-18	USA	KP135187	KP135258	Floudas and Hibbett, 2015
*Phanerochaete rhodella*	Miettinen 17278	USA	KU893882	—	Spirin et al., 2017
*Phanerochaete sanguinea*	HHB-7524	USA	KP135101	KP135244	Floudas and Hibbett, 2015
*Phanerochaete sanguinea*	Niemela 7993	Finland	KP135105	—	Floudas and Hibbett, 2015
*Phanerochaete sanguineocarnosa*	FD-359	USA	KP135122	KP135245	Floudas and Hibbett, 2015
*Phanerochaete sanguineocarnosa*	FD-528	USA	KP135121	—	Floudas and Hibbett, 2015
*Phanerochaete sinensis*	GC 1809-56	China	MT235689	MT248176	Xu Y. L. et al., 2020
*Phanerochaete sinensis*	He 4660	China	MT235688	MT248175	Xu Y. L. et al., 2020
*Phanerochaete sordida*	FD-241	USA	KP135136	KP135252	Floudas and Hibbett, 2015
*Phanerochaete sordida*	GC 1708-162	China	MZ422828	MZ637212	Chen et al., 2021
*Phanerochaete* sp.	GC 1710-52	Vietnam	MZ422832	MZ637215	Chen et al., 2021
*Phanerochaete* sp.	Wu 0805-86	China	MZ422835	MZ637218	Chen et al., 2021
***Phanerochaete subsanguinea***	**CLZhao 10470**	**China**	**MZ435348**	**MZ435352**	**Wang and Zhao**, **2021**
***Phanerochaete subsanguinea***	**CLZhao 10477** ^*^	**China**	**MZ435349**	**MZ435353**	**Wang and Zhao**, **2021**
***Phanerochaete subsanguinea***	**He 6131**	**China**	**ON964009**	**ON963990**	**Present study**
*Phanerochaete taiwaniana*	Wu 0112-13	China	MF399412	GQ470665	Wu et al., 2010, 2018a
*Phanerochaete taiwaniana*	Wu 880824-17	China	MZ422842	GQ470666	Wu et al., 2010; Chen et al., 2021
*Phanerochaete velutina*	GC 1604-56	China	MZ422844	MZ637224	Chen et al., 2021
*Phanerochaete velutina*	HHB-15343	USA	KP135184	—	Floudas and Hibbett, 2015
“*Phlebia” firma*	Edman K268	Sweden	EU118654	EU118654	Larsson, 2007
*Phlebiopsis alba*	GC 1508-110	China	MZ637042	MZ637246	Chen et al., 2021
*Phlebiopsis alba*	GC 1708-20	China	MZ637043	MZ637247	Chen et al., 2021
*Phlebiopsis albescens*	He 5805	Sri Lanka	MT452526	—	Zhao et al., 2021
*Phlebiopsis amethystea*	URM 84741	Brazil	MK993645	MK993639	Lima et al., 2020
*Phlebiopsis amethystea*	URM 93248	Brazil	MK993644	MK993638	Lima et al., 2020
*Phlebiopsis brunnea*	He 5822	Sri Lanka	MT452527	MT447451	Zhao et al., 2021
*Phlebiopsis brunneocystidiata*	Chen 666	China	MT561707	GQ470640	Wu et al., 2010; Chen et al., 2021
*Phlebiopsis brunneocystidiata*	Chen 1143	China	MZ637048	MZ637249	Chen et al., 2021
***Phlebiopsis cana***	**He 5728**	**Sri Lanka**	**—**	**ON963991**	**Present study**
***Phlebiopsis cana***	**He 5958** ^*^	**China**	**—**	**ON963992**	**Present study**
*Phlebiopsis castanea*	GC 1612-6	China	KY688208	MZ637250	Chen et al., 2018a, 2021
*Phlebiopsis castanea*	Spirin 5295	Russia	KX752610	KX752610	Miettinen et al., 2016
*Phlebiopsis* cf. *dregeana*	SFC 980804-4	Korea	AF479669	—	Lim and Jung, 2003
*Phlebiopsis* cf. *dregeana*	UOC-DAMIA-D46	Sri Lanka	KP734203	—	Unpublished
*Phlebiopsis crassa*	GC 1602-45	China	MZ637049	MZ637251	Chen et al., 2021
*Phlebiopsis crassa*	KKN-86-Sp	USA	KP135394	KP135215	Floudas and Hibbett, 2015
*Phlebiopsis cylindrospora*	He 5932	China	—	MT447444	Zhao et al., 2021
*Phlebiopsis cylindrospora*	He 5984	China	—	MT447445	Zhao et al., 2021
*Phlebiopsis darjeelingensis*	GC 1409-20	China	MZ637052	MZ637252	Chen et al., 2021
*Phlebiopsis darjeelingensis*	GC 1508-8	China	MZ637053	MZ637253	Chen et al., 2021
*Phlebiopsis flavidoalba*	GC 1807-47	USA	MZ637050	MZ637254	Chen et al., 2021
*Phlebiopsis flavidoalba*	FD-263	USA	KP135402	KP135271	Floudas and Hibbett, 2015
*Phlebiopsis friesii*	He 5722	Sri Lanka	MT452528	MT447413	Zhao et al., 2021
*Phlebiopsis friesii*	He 5817	Sri Lanka	MT452529	MT447414	Zhao et al., 2021
*Phlebiopsis galochroa*	FP-102937-Sp	USA	KP135391	KP135270	Floudas and Hibbett, 2015
*Phlebiopsis gigantea*	FCUG 1417	Norway	MZ637051	AF141634	Parmasto and Hallenberg, 2000; Chen et al., 2021
*Phlebiopsis gigantea*	FP-70857-Sp	USA	KP135390	KP135272	Floudas and Hibbett, 2015
*Phlebiopsis griseofuscescens*	CLZhao 3692	China	MT180946	MT180950	Xu T. M. et al., 2020
*Phlebiopsis griseofuscescens*	He 5734	Sri Lanka	MT561708	MT598032	Zhao et al., 2021
*Phlebiopsis laxa*	Wu 9311-17	China	MT561710	GQ470649	Wu et al., 2010; Chen et al., 2021
*Phlebiopsis magnicystidiata*	He 5648	China	MT386377	MT447409	Zhao et al., 2021
*Phlebiopsis magnicystidiata*	Wu 890805-1	China	MT561711	GQ470667	Zhao et al., 2021
*Phlebiopsis membranacea*	He 3849	China	MT386401	MT447441	Zhao et al., 2021
*Phlebiopsis membranacea*	He 6062	China	MT386402	MT447442	Zhao et al., 2021
*Phlebiopsis odontoidea*	GC 1708-181	China	MZ637054	MZ637255	Chen et al., 2021
*Phlebiopsis odontoidea*	GC 1708-182	China	MZ637055	MZ637256	Chen et al., 2021
*Phlebiopsis pilatii*	Spirin 5048	Russia	KX752590	KX752590	Miettinen et al., 2016
*Phlebiopsis pilatii*	Wu 1707-18	China	MZ637056	MZ637257	Chen et al., 2021
*Phlebiopsis sinensis*	He 4673	China	MT386397	MT447435	Zhao et al., 2021
*Phlebiopsis sinensis*	He 5662	China	MT386398	MT447436	Zhao et al., 2021
*Phlebiopsis* sp.	GC 1705-63	China	MZ637058	MZ637258	Chen et al., 2021
*Phlebiopsis* sp.	GC 1807-3	USA	MZ637059	MZ637259	Chen et al., 2021
*Phlebiopsis* sp.	KHL13055	Costa Rica	EU118662	EU118662	Larsson, 2007
*Phlebiopsis xuefengensis*	JZou5501	China	MT554931	MT554924	Li et al., 2021
*Phlebiopsis xuefengensis*	WS01	China	MT554921	MT554928	Li et al., 2021
*Phlebiopsis yunnanensis*	CLZhao 3958	China	MH744140	MH744142	Zhao et al., 2019
*Phlebiopsis yunnanensis*	GC 1708-169	China	MZ637060	MZ637260	Chen et al., 2021
*Phlebiopsis yushaniae*	Chen 2358	China	MZ637047	MZ637261	Chen et al., 2021
*Pirex concentricus*	OSC-41587	USA	KP134984	KP135275	Floudas and Hibbett, 2015
*Quasiphlebia densa*	HHB-12357	USA	MZ637065	MZ637264	Chen et al., 2021
*Quasiphlebia densa*	WEI 17-057	China	MZ637066	MZ637265	Chen et al., 2021
*Rhizochaete americana*	FP-102188	USA	KP135409	KP135277	Floudas and Hibbett, 2015
*Rhizochaete americana*	HHB-2004	USA	AY219391	AY219391	Greslebin et al., 2004
*Rhizochaete belizensis*	FP-150712	Belize	KP135408	KP135280	Floudas and Hibbett, 2015
*Rhizochaete borneensis*	WEI 16-426	China	MZ637070	MZ637270	Chen et al., 2021
*Rhizochaete brunnea*	MR11455	Argentina	AY219389	AY219389	Greslebin et al., 2004
*Rhizochaete chinensis*	Wu 0910-45	China	LC387335	MF110294	Chen et al., 2018b; Wu et al., 2018b
*Rhizochaete chinensis*	Wu 0910-59	China	MZ637071	MZ637271	Chen et al., 2021
*Rhizochaete filamentosa*	FP-105240	USA	KP135411	AY219393	Floudas and Hibbett, 2015
*Rhizochaete filamentosa*	HHB-3169	USA	KP135410	KP135278	Floudas and Hibbett, 2015
*Rhizochaete flava*	PR1141	USA	KY273030	KY273033	Nakasone et al., 2017
*Rhizochaete fissurata*	CLZhao 7965	China	MZ713641	MZ713845	Gu and Zhao, 2021
*Rhizochaete fissurata*	CLZhao 10407	China	MZ713642	MZ713846	Gu and Zhao, 2021
*Rhizochaete fissurata*	He 2336	China	ON964017	—	Present study
*Rhizochaete fissurata*	He 6186	China	ON964018	ON964000	Present study
*Rhizochaete fissurata*	He 6259	China	ON964019	ON964001	Present study
*Rhizochaete fouquieriae*	KKN-121	USA	AY219390	GU187608	Greslebin et al., 2004; Binder et al., 2010
*Rhizochaete grandinosa*	CLZhao 3117	China	MZ713644	MZ713848	Gu and Zhao, 2021
*Rhizochaete lutea*	Wu 880417-5	China	MZ637072	GQ470651	Wu et al., 2010; Chen et al., 2021
*Rhizochaete lutea*	He 5964	China	**—**	ON964008	Present study
***Rhizochaete nakasoneae***	**He 2291** ^*^	**China**	**ON964016**	**ON963998**	**Present study**
***Rhizochaete nakasoneae***	**He 4821**	**China**	**—**	**ON963999**	**Present study**
***Rhizochaete nakasoneae***	**WEI 16-383**	**China**	**MZ637073**	**MZ637272**	**Chen et al.**, **2021**
***Rhizochaete nakasoneae***	**Wu 1008-62**	**China**	**MZ637074**	**MZ637273**	**Chen et al.**, **2021**
*Rhizochaete radicata*	FD-123	USA	KP135407	KP135279	Floudas and Hibbett, 2015
*Rhizochaete radicata*	HHB-1909	USA	AY219392	AY219392	Greslebin et al., 2004
*Rhizochaete* sp.	He 4628	China	ON964021	**—**	Present study
***Rhizochaete subradicata***	**He 2377**	**China**	**ON964023**	**ON964004**	**Present study**
***Rhizochaete subradicata***	**He 3213** ^*^	**China**	**OP102693**	**OP102695**	**Present study**
***Rhizochaete subradicata***	**He 4282**	**China**	**ON964025**	**ON964006**	**Present study**
***Rhizochaete subradicata***	**He 5561**	**China**	**ON964026**	**ON964007**	**Present study**
*Rhizochaete sulphurina*	HHB-5604	USA	KY273031	GU187610	Binder et al., 2010; Nakasone et al., 2017
*Rhizochaete sulphurosa*	URM87190	Brazil	KT003522	KT003519	Chikowski et al., 2016
***Rhizochaete terrestris***	**He 7713**	**China**	**OP549029**	**OP549030**	**Present study**
***Rhizochaete terrestris***	**He 6694**	**China**	**ON964022**	**ON964003**	**Present study**
*Rhizochaete violascens*	KHL11169	Norway	**—**	EU118612	Larsson, 2007
***Rhizochaete yunnanensis***	**He 3302** ^*^	**China**	**ON964020**	**ON964002**	**Present study**
***Roseograndinia aurantiaca***	**CLZhao 10480**	**China**	**MW209022**	**MW209011**	**Wang and Zhao**, **2020**
***Roseograndinia aurantiaca***	**CLZhao 10487** ^*^	**China**	**MW209023**	**MW209012**	**Wang and Zhao**, **2020**
***Roseograndinia aurantiaca***	**Wu 1307-132**	**China**	**MZ637076**	**MZ637274**	**Chen et al.**, **2021**
***Roseograndinia aurantiaca***	**Wu 1307-137**	**China**	**MZ637077**	**MZ637275**	**Chen et al.**, **2021**
*Roseograndinia minispora*	WEI 18-508	China	MZ637078	MZ637276	Chen et al., 2021
*Roseograndinia minispora*	WEI 18-511	China	MZ637079	MZ637277	Chen et al., 2021
*Roseograndinia minispora*	He 5808	Sri Lanka	ON964015	—	Present study
***Roseograndinia zixishanensis***	**CLZhao 7206**	**China**	**MZ305280**	**MZ305289**	**Wang et al.**, **2021**
***Roseograndinia zixishanensis***	**CLZhao 7718** ^*^	**China**	**MZ305285**	**MZ305293**	**Wang et al.**, **2021**

New taxa are set in bold with type specimens indicated with an asterisk (^*^).

### Phylogenetic analyses

Four separate datasets of concatenated ITS-nrLSU sequences of the *Donkia, Phlebiopsis, Rhizochaete*, and *Phanerochaete* clades in the Phanerochaeteaceae were analyzed. The clades recognition and ingroup and outgroup taxa selections were mainly discussed in the study by Chen et al. (2021). The *Phanerochaete* clade included only taxa in the core group of the genus. *Rhizochaete radicata* (Henn.) Gresl., Nakasone & Rajchenb. was selected as the outgroup for the *Donkia* and *Phlebiopsis* clades, while *Phlebiopsis gigantea* (Fr.) Jülich was used as the outgroup for the *Rhizochaete* and *Phanerochaete* clades. The ITS and nrLSU sequences were aligned separately using MAFFT v.7^4^ (Katoh et al., 2017) with the G-INS-I iterative refinement algorithm and optimized manually in BioEdit v.7.0.5.3. Then, the separate alignments were concatenated using Mesquite v.3.5.1 (Maddison and Maddison, 2018).

Maximum parsimony (MP), maximum likelihood (ML) analyses, and Bayesian inference (BI) were carried out using PAUP^*^ v.4.0b10 (Swofford, 2002), RAxML v.8.2.10 (Stamatakis, 2014), and MrBayes 3.2.6 (Ronquist et al., 2012), respectively. In MP analysis, trees were generated using 100 replicates of random stepwise addition of sequence and the tree-bisection reconnection (TBR) branch-swapping algorithm with all characters given equal weight. Branch supports for all parsimony analyses were estimated by performing 1,000 bootstrap replicates with a heuristic search of 10 random-addition replicates for each bootstrap replicate. In ML analysis, statistical support values were obtained using rapid bootstrapping with 1,000 replicates, with default settings for other parameters. For BI, the best-fit substitution model was estimated with jModeltest v.2.17 (Darriba et al., 2012). Four Markov chains were run for 1,000,000 for the three datasets of the *Donkia, Phanerochaete*, and *Phlebiopsis* clades and the 2,000,000 for the dataset of the *Rhizochaete* clade until the split deviation frequency value was lower than 0.01. Trees were sampled every 100th generation. The first quarter of the trees, which represented the burn-in phase of the analyses, were discarded, and the remaining trees were used to calculate posterior probabilities (BPP) in the majority rule consensus tree.

## Results

### Phylogenetic analyses

The dataset of the *Donkia* clade contained 39 ITS and 38 nrLSU sequences from 39 samples, representing 19 ingroup taxa and the outgroup (Table 1), and had an aligned length of 2,009 characters, of which 251 were parsimony-informative. MP analysis yielded two equally parsimonious trees (TL = 899, CI = 0.617, RI = 0.793, RC = 0.489, HI = 0.383). The dataset of *Phlebiopsis* clade contained 45 ITS and 46 nrLSU sequences from 49 samples, representing 28 ingroup taxa and the outgroup, and had an aligned length of 2,006 characters, of which 224 were parsimony-informative. MP analysis yielded 17 equally parsimonious trees (TL = 862, CI = 0.542, RI = 0.770, RC = 0.417, HI = 0.458). The dataset of the *Rhizochaete* clade contained 34 ITS and 35 nrLSU sequences from 37 samples, representing 20 ingroup taxa and the outgroup, and had an aligned length of 2,164 characters, of which 295 were parsimony-informative. MP analysis yielded nine equally parsimonious trees (TL = 1,150, CI = 0.572, RI = 0.703, RC = 0.402, HI = 0.428). The dataset of the *Phanerochaete* clade contained 42 ITS and 33 nrLSU sequences from 42 samples, representing 24 ingroup taxa and the outgroup, and had an aligned length of 2,065 characters, of which 275 were parsimony-informative. MP analysis yielded 19 equally parsimonious trees (TL = 885, CI = 0.591, RI = 0.745, RC = 0.440, HI = 0.409).

**Figure 1:**
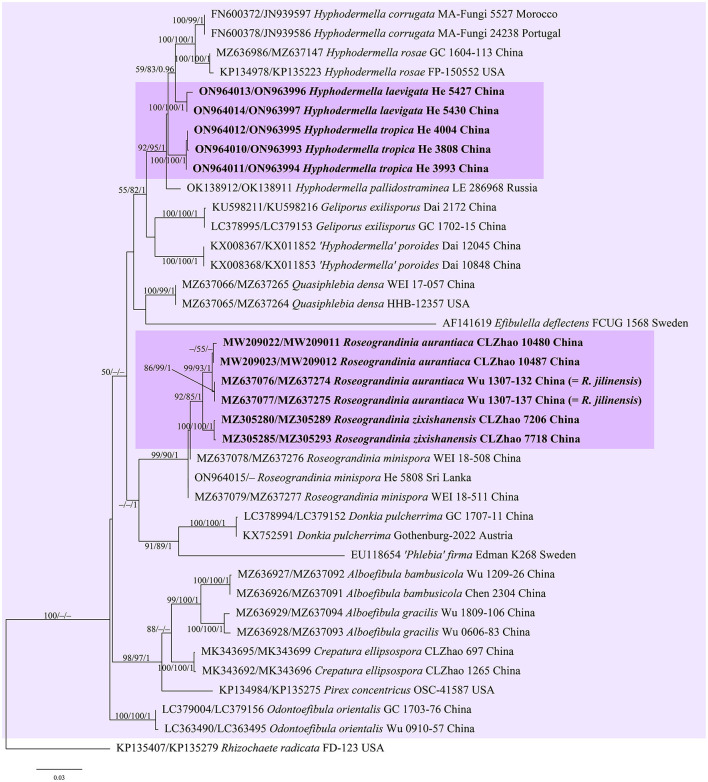
Phylogenetic tree obtained from ML analysis of the ITS-nrLSU sequences of the *Donkia* clade. Branches are labeled with parsimony bootstrap values (≥50%, first), likelihood bootstrap values (≥50%, second), and Bayesian posterior probabilities (≥0.95, third). New taxa are set in bold and highlighted.

**Figure 2:**
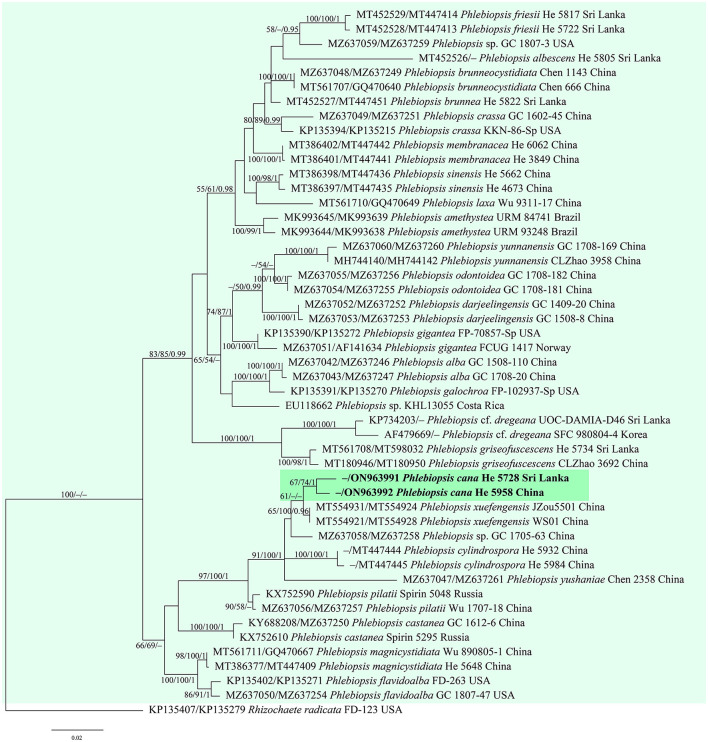
Phylogenetic tree obtained from ML analysis of the ITS-nrLSU sequences of *Phlebiopsis*. Branches are labeled with parsimony bootstrap values (≥50%, first), likelihood bootstrap values (≥50%, second), and Bayesian posterior probabilities (≥0.95, third). The new species are set in bold and highlighted.

**Figure 3:**
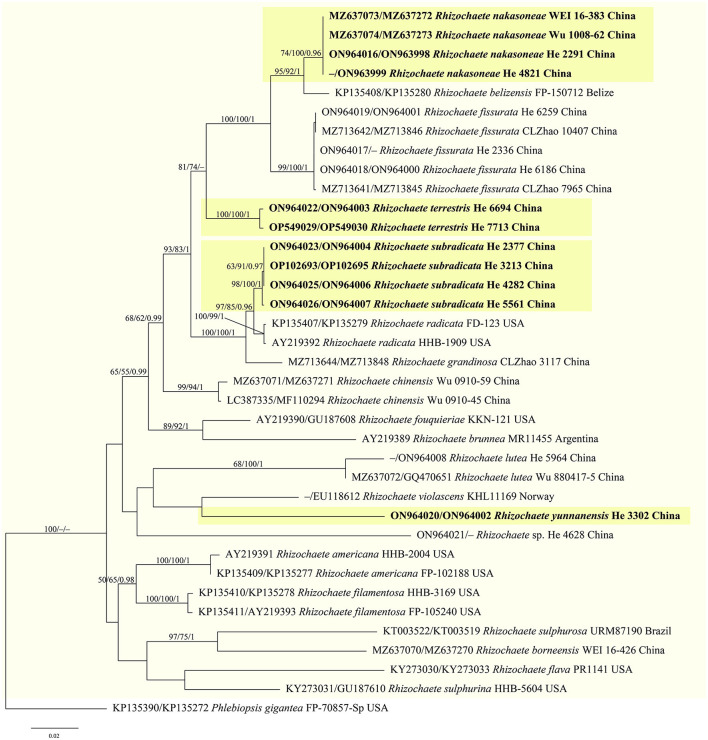
Phylogenetic tree obtained from ML analysis of the ITS-nrLSU sequences of *Rhizochaete*. Branches are labeled with parsimony bootstrap values (≥50%, first), likelihood bootstrap values (≥50%, second), and Bayesian posterior probabilities (≥0.95, third). New species are set in bold and highlighted.

**Figure 4:**
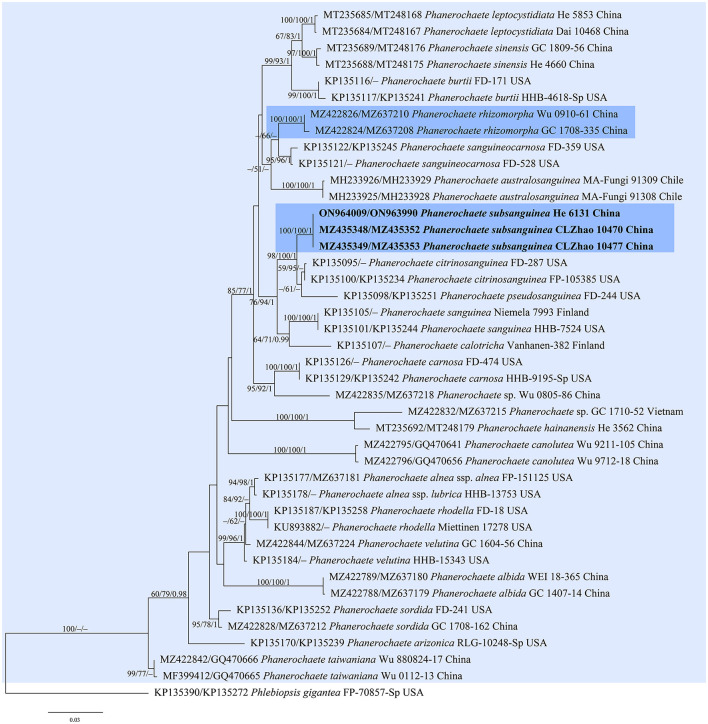
Phylogenetic tree obtained from ML analysis of the ITS-nrLSU sequences of *Phanerochaete*. Branches are labeled with parsimony bootstrap values (≥50%, first), likelihood bootstrap values (≥50%, second), and Bayesian posterior probabilities (≥0.95, third). The new replacement names are set in bold and highlighted.

jModelTest suggested GTR+I+G as the best-fit models of nucleotide evolution for the four datasets. The average standard deviations of the split frequencies of BI were 0.007436, 0.007608, 0.007786, and 0.006954 for the *Donkia, Phlebiopsis, Rhizochaete*, and *Phanerochaete* clades, respectively, at the end of the runs. The MP and BI analyses resulted in almost identical tree topologies with the ML analysis. Only the ML trees of the four clades are shown in Figures 1–4 with the parsimony bootstrap values (≥50%, first), the likelihood bootstrap values (≥50%, second), and Bayesian posterior probabilities (≥0.95, third) labeled along the branches.

In the trees (Figures 1–4), seven new species, *Hyphodermella laevigata, H. tropica, Phlebiopsis cana, Rhizochaete nakasoneae, R. subradicata, R. terrestris*, and *R. yunnanensis*, formed distinct lineages. In our tree of the *Donkia* clade (Figure 1), *Hyphodermella* s.s. and *Roseograndinia* were strongly supported as separate genera (92/95/1 and 99/90/1). Meanwhile, *H. zixishanensis* C.L. Zhao and *H. aurantiaca* C.L. Zhao that is conspecific with *R. jilinensis* C.C. Chen & Sheng H. Wu were nested within *Roseograndinia*. In the *Phanerochaete* clade (Figure 4), the new names *P. subsanguinea* (= *P. rhizomorpha* C.L. Zhao & D.Q. Wang) and *P. rhizomorpha* (C.C. Chen, Sheng H. Wu & S.H. He) formed two distinct lineages.

### Taxonomy

#### *Hyphodermella laevigata* Yue Li & S.H. He, sp. nov.

MycoBank: MB846336

Diagnosis—The species is recognized by a smooth hymenophore, the absence of cystidia and cystidioid hyphal ends, and small ellipsoid basidiospores.

Type—China, Guizhou Province, Chishui County, Suoluo Nature Reserve, on a dead angiosperm branch, 7 July 2018, He 5427 (BJFC 026488, holotype).

Etymology—Refer to the smooth hymenophore.

Fruiting body—Basidiomes annual, resupinate, widely effused, closely adnate, inseparable from substrate, and membranaceous, first as small patches, later confluent up to 10 cm long, 2 cm wide, and up to 80 μm thick in section. Hymenophore smooth, pale yellow (4A3) to grayish yellow (4B3), slightly darkening in KOH, and not cracked; margin thinning out, determinate, adnate, fimbriate, and concolorous with hymenophore surface; and context is pale yellow.

Microscopic structures—Hyphal system monomitic; generative hyphae simple-septate. Subiculum distinct; hyphae colorless, thick-walled, smooth, frequently branched, moderately septate, loosely interwoven, and 2.5–6.5 μm in diameter. Subhymenium thin; hyphae colorless, thin-walled, smooth, infrequently branched, moderately septate, more or less vertical, and 1.5–2.5 μm in diameter. Cystidia absent. Basidia clavate to subcylindrical, colorless, thin-walled, smooth, with a basal simple septum and four sterigmata, and 10–30 × 5–7 μm; basidioles similar to basidia, but smaller. Basidiospores ellipsoid, with an apiculus, usually with one or two oil drops, colorless, thin-walled, smooth, (4.6–) 4.8–6 (−6.3) × 3–3.8 (−4.1) μm, L = 5.3 μm, W = 3.5 μm, and Q = 1.46–1.56 (*n* = 60/2).

Additional specimens examined—China, Guizhou Province, Chishui County, Suoluo Nature Reserve, on a dead angiosperm branch, 7 July 2018, He 5430 (BJFC 026491).

**Figure 5:**
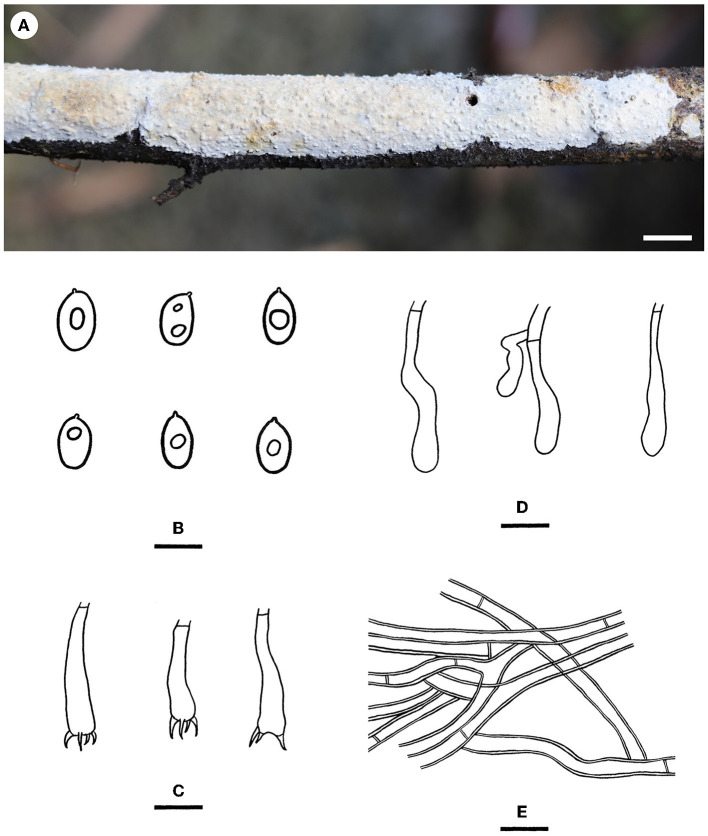
*Hyphodermella laevigata* [from the holotype He 5427; scale bars: **(A)** = 1 cm; **(B–E)** = 10 μm]. **(A)** Basidiomes; **(B)** Basidiospores; **(C)** Basidia; **(D)** Basidioles; **(E)** Hyphae from subiculum.

Notes—*Hyphodermella laevigata* (Figure 5) is characterized by membranaceous basidiomes with smooth hymenophores, the absence of cystidia and cystidioid hyphal ends, and small ellipsoid basidiospores. In the phylogenetic tree (Figure 1), *H. laevigata* formed a distinct lineage sister to *H. corrugata* (Fr.) J. Erikss. & Ryvarden and *H. rosae* (Bres.) Nakasone, which have grandinioid or odontioid hymenophores, encrusted hyphal ends projecting beyond the hymenium and obviously larger basidiospores (8–10 × 5–7 μm of *H. corrugata*, 7–8.5 × 4.5–5.5 μm of *H. rosae*, Bernicchia and Gorjón, 2010). *Hyphodermella maunakeaensis* Gilb. & Hemmes from Hawaii is similar to *H. laevigata* by sharing a loose texture and relatively small basidiospores but differs in having a finely hydnaceous hymenophore with projecting fascicles of encrusted hyphoid cystidia and slightly longer basidiospores (6.5–7.5 μm, Gilbertson, 2001).

#### *Hyphodermella tropica* Yue Li & S.H. He, sp. nov.

MycoBank: MB846337

Diagnosis—The species is recognized by a grandinioid hymenophore, the presence of encrusted cystidioid hyphal ends, and broad ellipsoid to ovoid basidiospores.

Type—China, Hainan Province, Baoting County, Qixianling Forest Park, on a dead angiosperm branch, 11 June 2016, He 3993 (BJFC 022495, holotype, CFMR, isotype).

Etymology—Refer to the known distribution in tropic China.

Fruiting body—Basidiomes annual, resupinate, widely effused, closely adnate, inseparable from substrate, and coriaceous, first as small patches and later confluent up to 11 cm long, 3 cm wide, and up to 80 μm thick in section. Hymenophore grandinioid, grayish orange (5B4) to brownish orange [5C (4–5)], darkening in KOH, and not cracked upon drying; the margin thinning out, adnate, indistinct, and concolorous with hymenophore surface; and context is grayish orange.

Microscopic structures—Hyphal system monomitic; generative hyphae simple-septate. Subiculum distinct; hyphae colorless, thick-walled, smooth, frequently branched, moderately septate, more or less parallel to the substrate, and 2–4 μm in diameter. Subhymenium distinct, thickening, composed of encrusted cystidioid hyphal ends, and hyphae with masses of crystals; hyphae colorless, slightly thick-walled, smooth, moderately branched and septate, loosely interwoven, and 1.5–3 μm in diameter. Cystidioid hyphal ends present, subcylindrical, colorless, thick-walled, heavily encrusted, mostly embedded, and 20–50 × 6–10 μm (crystals included). Basidia subcylindrical, colorless, thin-walled, smooth, usually with oil drops, with a basal simple septum and four sterigmata, and 15–27 × 4–8 μm; basidioles in shape similar to basidia, but slightly smaller. Basidiospores which are broadly ellipsoid to ovoid, with an apiculus, usually with oil drops, colorless, thin-walled, smooth, IKI–, CB–, 5–6 (−6.5) × (3–) 4–5 (−5.5) μm, L = 5.7 μm, W = 4.4 μm, and Q = 1.23–1.34 (*n* = 60/2).

Additional specimens examined—China, Guizhou Province, Libo County, Maolan Nature Reserve, on a dead angiosperm branch, 16 June 2016, He 3808 (BJFC 022307, CFMR); Hainan Province, Baoting County, Qixianling Forest Park, on a dead angiosperm branch, 11 June 2016, He 4004 (BJFC 022506, CFMR).

**Figure 6:**
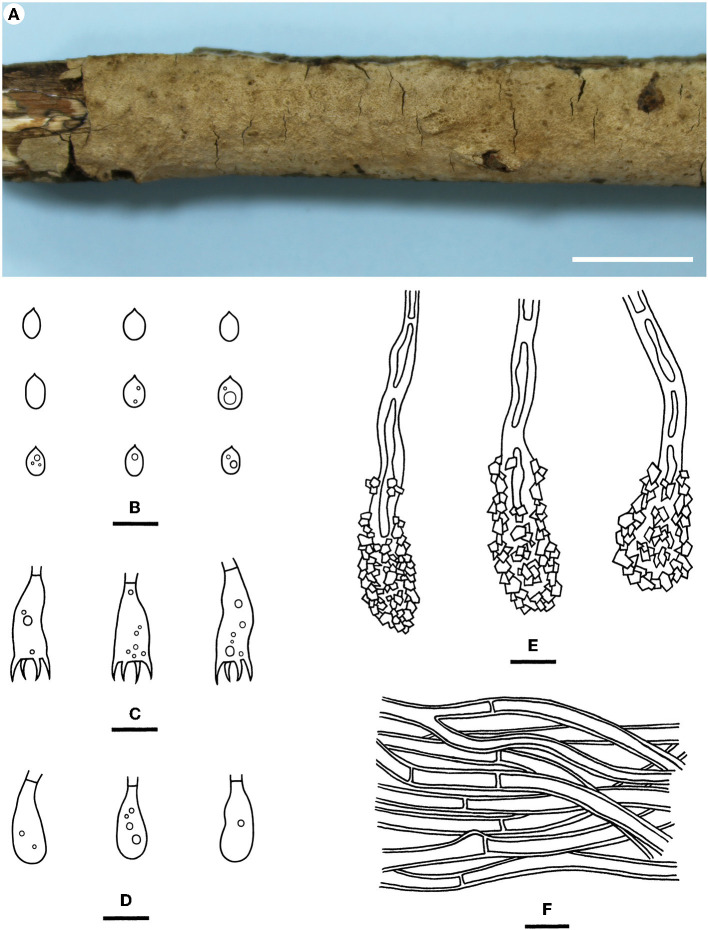
*Hyphodermella tropica* [from the holotype He 3933; scale bars: **(A)** = 1 cm; **(B–F)** = 10 μm]. **(A)** Basidiomes; **(B)** Basidiospores; **(C)** Basidia; **(D)** Basidioles; **(E)** Encrusted cystidioid hyphal ends; **(F)** Hyphae from subiculum.

Notes—*Hyphodermella tropica* (Figure 6) is characterized by coriaceous basidiomes with a grandinioid hymenophore, the presence of encrusted cystidioid hyphal ends, and broadly ellipsoid to ovoid basidiospores. In the phylogenetic tree (Figure 1), *H. tropica* formed a distinct lineage sister to *H. pallidostraminea* Bukharova & Volobuev and *H. laevigata*. *Hyphodermella pallidostraminea* from Russia differs from *H. tropica* by having a smooth to slightly tuberculate hymenophore, not encrusted cystidioid hyphal ends in hymenium, and narrower ellipsoid basidiospores (3–3.5 μm, Crous et al., 2021). *Hyphodermella laevigata* can be easily distinguished from *H. tropica* by the smooth hymenophore, the loose texture of the subiculum, and the lack of encrusted cystidioid hyphal ends. *Hyphodermella brunneocontexta* Duhem & Buyck from France is similar to *H. tropica* by sharing an odontioid hymenophore, encrusted cystidioid hyphal ends, and ellipsoid to ovoid basidiospores but differs in having a brown context with densely interwoven hyphae (Duhem and Buyck, 2011).

#### *Roseograndinia aurantiaca* (C.L. Zhao) Yue Li & S.H. He, comb. nov.

MycoBank: MB846338

= *Hyphodermella aurantiaca* C.L. Zhao, Annales Botanici Fennici 58: 65, 2020. [MB#837949]

= *Roseograndinia jilinensis* C.C. Chen & Sheng H. Wu, Fungal Diversity 111: 396, 2021. [MB#840765]

Notes—Both *H. aurantiaca* and *R. jilinensis* were recently described from China, and are similar to each other by sharing a smooth to tuberculate, reddish hymenophore, a compact context with a thickened subhymenium, the absence of cystidia, and relatively small ellipsoid basidiospores but differ in the size of the basidiospores according to the descriptions (3–4 × 2–2.8 μm of *H. aurantiaca* vs. 4.6–5.5 × 2.6–3.1 μm of *R. jilinensis*, Wang and Zhao, 2020; Chen et al., 2021). However, in our phylogenetic tree (Figure 1), the types of the two species clustered in a strongly supported lineage in the *Roseograndinia* clade (99/93/1) with the ITS sequence similarity reaching 99.5% (3 differences of 600 base pairs). Thus, based on the morphological and molecular evidence, the new combination *R. aurantiaca* is proposed herein, since the epithet “*aurantiaca*” has the priority.

#### *Roseograndinia zixishanensis* (C.L. Zhao) Yue Li & S.H. He, comb. nov.

MycoBank: MB846339

= *Hyphodermella zixishanensis* C.L. Zhao, Nordic Journal of Botany 39 (8): e03329, 4, 2021. [MB#839869]

Notes—The species *H. zixishanensis* was recently described from Yunnan Province, southwestern China, based on morphological and molecular evidence (Wang et al., 2021). In our phylogenetic tree based on a more complete sampling, including many newly described taxa (Figure 1), the species was nested within the *Roseograndinia* clade, which was strongly supported as a monophyletic clade with three species and independent of *Hyphodermella*. Morphologically, *Roseograndinia zixishanensis* has the typical characteristics of the genus by possessing ceraceous basidiomes with a smooth to tuberculate hymenophore, a monomitic hyphal system with simple-septate generative hyphae, and a lack of cystidia (Chen et al., 2021; Wang et al., 2021).

#### *Phlebiopsis cana* Yue Li & S.H. He, sp. nov.

MycoBank: MB846340

Diagnosis—The species is recognized by a smooth hymenophore with a gray to brownish gray hymenial surface, the presence of short lamprocystidia and short cylindrical basidiospores, and its growth on bamboo.

Type—China, Hainan Province, Lingshui County, Diaoluoshan Nature Reserve, on a culm of dead bamboo, 2 July 2019, He 5958 (BJFC 030834, holotype).

Etymology—Refer to the gray color of basidiomes.

Fruiting body—Basidiomes annual, resupinate, widely effused, closely adnate, inseparable from the substrate, and coriaceous, first as small patches and later confluent up to 11 cm long, 2 cm wide, and up to 40 μm thick in section. Hymenophore smooth, gray (4C1) to brownish gray (4D2), slightly darkening in KOH, and not cracked upon drying; the margin thinning out, adnate, indistinct, and concolorous with hymenophore surface when juvenile, and darkening with age; and context is gray.

Microscopic structures—Hyphal system monomitic; generative hyphae simple-septate. Subiculum distinct, a compact texture, pale yellowish-brown, and up to 20 μm thick; hyphae colorless to pale yellow, thick-walled, smooth, rarely branched, moderately septate, more or less parallel to the substrate, slightly agglutinated, and 3–4 μm in diameter. Subhymenium indistinct; hyphae colorless, slightly thick-walled, rarely branched, moderately septate, densely interwoven, and 2–3 μm in diameter. Lamprocystidia scattered, subcylindrical to subfusiform, colorless, thick-walled, apically encrusted with small crystals, projecting beyond the hymenium up to 10 μm, and 15–30 × 5–7 μm (crystals included). Basidia clavate, colorless, thin-walled, smooth, with a basal simple septum and four sterigmata, and 12–18 × 3.5–5 μm; basidioles in shape similar to basidia, but slightly smaller. Basidiospores short cylindrical, with an apiculus, colorless, thin-walled, smooth, IKI–, CB–, (4.6–) 4.8–5.6 (−5.8) × 1.8–2 (−2.2) μm, L = 5.4 μm, W = 2 μm, and Q = 2.66–2.77 (*n* = 60/2).

Additional specimen examined—Sri Lanka, Western Province, Ingiriya, Dombagaskanda Forest Reserve, on a culm dead bamboo, 27 February 2019, He 5728 (BJFC 030595).

**Figure 7:**
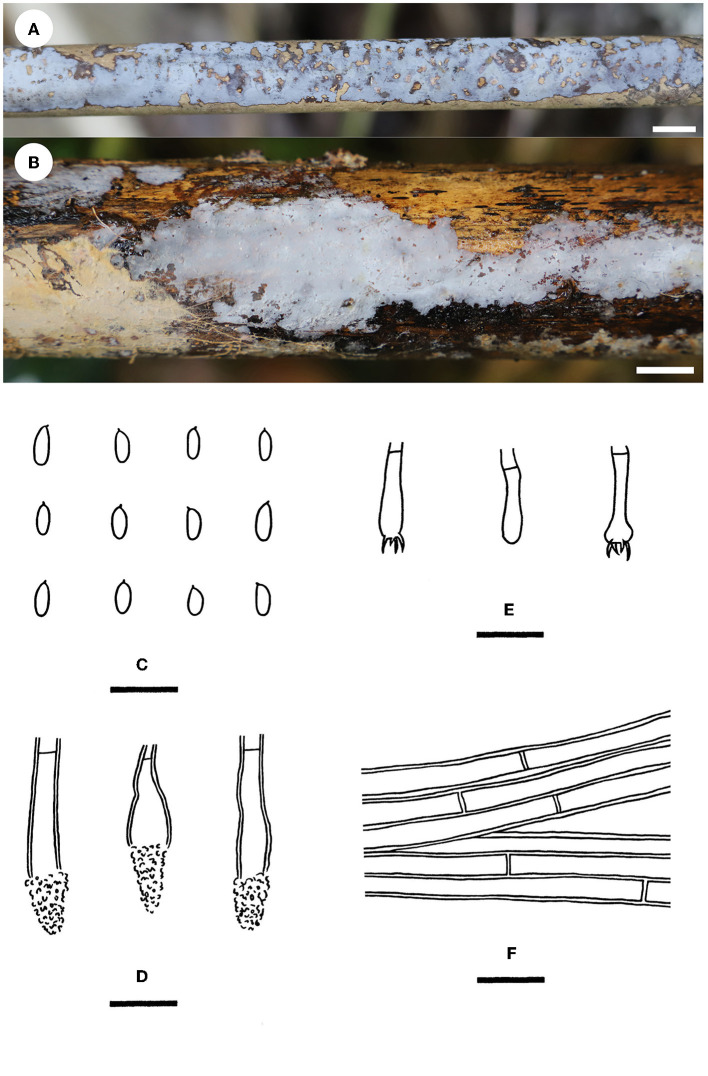
*Phlebiopsis cana* [**(A, C–F)** from the holotype He 5958; **(B)** from He 5728; scale bars: **(A, B)** = 1 cm; **(C–F)** = 10 μm]. **(A, B)** Basidiomes; **(C)** Basidiospores; **(D)** Basidia and a basidiole; **(E)** Lamprocystidia; **(F)** Hyphae from subiculum.

Notes—*Phlebiopsis cana* (Figure 7) is characterized by having thin basidiomes with a gray, smooth hymenophore surface, short lamprocystidia, and cylindrical basidiospores. In the phylogenetic tree (Figure 2), *P. cana* formed a lineage sister to *P. xuefengensis* J. Zou, which is an endophyte of *Gastrodia elata* from China. The descriptions of *P. xuefengensis* were based on cultures and not standard or comparable with *P. cana* (Li et al., 2021). *Phlebiopsis cylindrospora* Y.N. Zhao & S.H. He that was also found on bamboos in China is similar to *P. cana* by sharing short lamprocystidia and cylindrical basidiospores but differs in having hymenophores turning purple in KOH, generative hyphae encrusted with yellow, resinous granules, and slightly larger basidiospores (5.5–7.5 × 1.8–2.8 μm, Zhao et al., 2021). Another *Phlebiopsis* species on bamboo from Taiwan, *P. yushaniae* C.C. Chen & Sheng H. Wu, differs from *P. cana* by possessing white to cream basidiomes, possessing broadly ellipsoid to subglobose basidiospores (6.9–7.9 × 5.2–6.2 μm), and possessing a lack of cystidia (Chen et al., 2021). Phylogenetically, *P. cana, P. cylindrospora*, and *P. yushaniae* are grouped together with strong support values but formed distinct lineages (Figure 2).

#### *Rhizochaete nakasoneae* Yue Li, C.C. Chen & S.H. He, sp. nov.

MycoBank: MB846341

Diagnosis—The species is recognized by a smooth hymenophore turning pinkish buff in KOH, the presence of thick-walled lamprocystidia, and broadly ellipsoid to ovoid basidiospores.

Type—China, Hunan Province, Zhangjiajie, Zhangjiajie Forest Park, on a fallen angiosperm trunk, 7 July 2015, He 2291 (BJFC 020746, holotype).

Etymology—Named to honor Dr. Karen K. Nakasone (CFMR, USA), who contributed much to the taxonomy and phylogeny of *Rhizochaete*.

Fruiting body—Basidiomes annual, resupinate, widely effused, loosely adnate, easily separated from substrate, and membranaceous to pellicular, first as small patches, later confluent up to 4 cm long, 2 cm wide, and up to 120 μm thick in section. Hymenophore smooth, light orange (6A5) to grayish orange (5B5), turning pinkish buff in KOH, not cracked upon drying; the margin thinning out, adnate, fimbriate, sterile, white when fresh, and becoming pale yellow upon drying; hyphal cords are present, brownish yellow, and turning pinkish buff in KOH; and context is pale yellow.

Microscopic structures—Hyphal system monomitic; generative hyphae simple-septate. Subiculum distinct; hyphae colorless, thin- to slightly thick-walled, usually encrusted with crystals, frequently branched and septate, loosely interwoven, and 3–5 μm in diameter. Subhymenium thickening, composed of lamprocystidia and hyphae; hyphae colorless, thin- to slightly thick-walled, smooth, vertically arranged, moderately branched, rarely septate, and 2–3 μm in diameter. Lamprocystidia numerous, metuloid, subfusiform, colorless, thick-walled, heavily encrusted with crystals, embedded or projecting beyond the hymenium up to 25 μm, and 34–50 × 8–14 μm (crystals included), with walls up to 1.5–2 μm. Basidia clavate, colorless, thin-walled, smooth, with a basal simple septum and four sterigmata, and 18–24 × 3.5–5 μm; basidioles in shape similar to basidia, but slightly smaller. Basidiospores broadly ellipsoid to ovoid, with an apiculus, colorless, thin-walled, smooth, usually with one or two oil drops, IKI–, CB–, 2.5–4 × (1.8–) 2–2.5 μm, L = 3.1 μm, W = 2.1 μm, and Q = 1.34–1.59 (*n* = 60/2).

Additional specimens examined—China, Guangxi Zhuang Autonomous Region, Xing'an County, Mao'ershan Nature Reserve, on an angiosperm stump, 13 July 2017, He 4821 (BJFC 024340, CFMR); Hunan Province, Zhangjiajie, Zhangjiajie Forest Park, on a rotten angiosperm trunk, 17 August 2010, Wu 1008-62 (TNM F0025327); Taiwan, Nantou County, Jenai Township, Aowanda National Forest Recreation Area, on a fallen angiosperm branch, 3 October 2016, WEI 16-383 (TNM F0031055).

**Figure 8:**
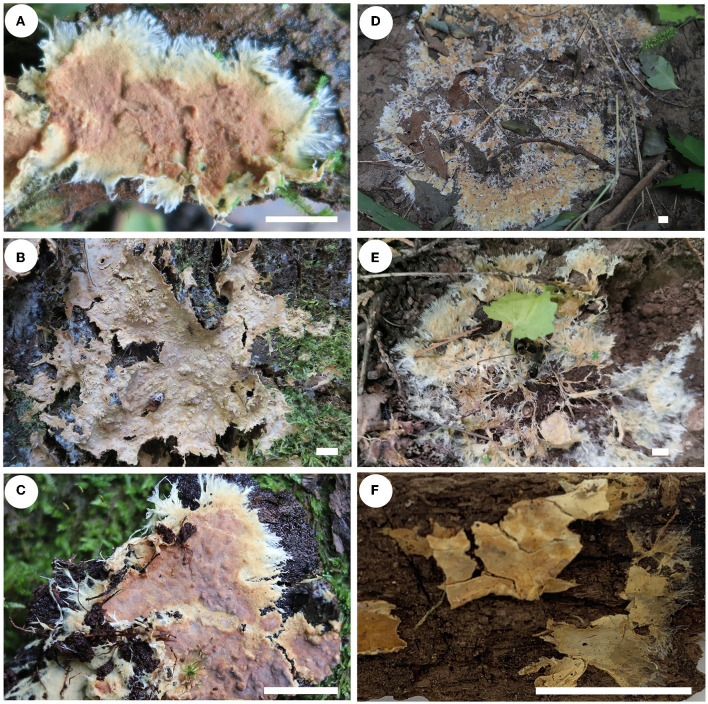
Basidiomes of *Rhizochaete* [scale bars: **(A–F)** = 1 cm]. **(A)** *R. nakasoneae* (He 4821, paratype); **(B, C)** *R. subradicata* [**(B)** He 3213, holotype; **(C)** He 5561, paratype]; **(D, E)** *R. terrestris* [**(D)** He 7713, holotype; **(E)** He 6694, paratype]; **(F)** *R. yunnanensis* (He 3302, holotype).

**Figure 9:**
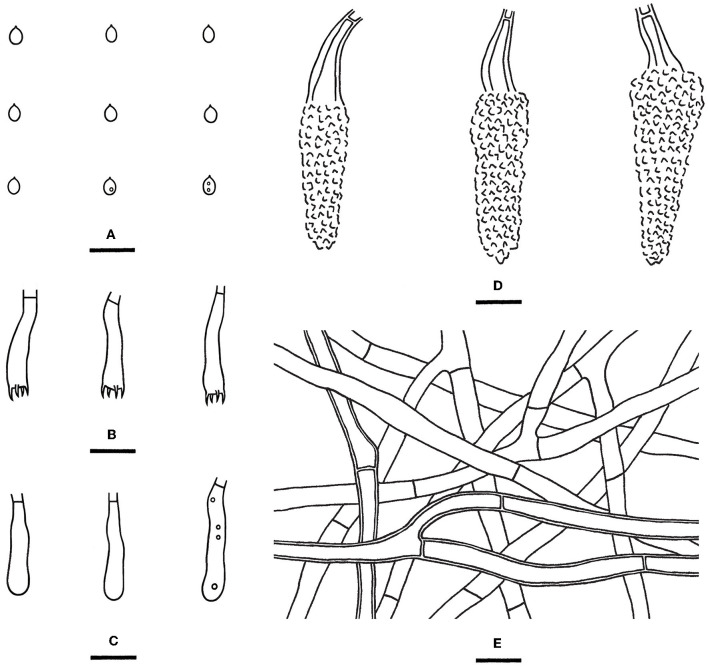
*Rhizochaete nakasoneae* [from the holotype He 2291; scale bars: **(A–E)** = 10 μm]. **(A)** Basidiospores; **(B)** Basidia; **(C)** Basidioles; **(D)** Lamprocystidia; **(E)** Hyphae from subiculum.

Notes—*Rhizochaete nakasoneae* (Figures 8A, 9) is characterized by the thin pellicular basidiomes, simple-septate generative hyphae, thick-walled subfusiform cystidia, and small, broadly ellipsoid to ovoid basidiospores. Morphologically and phylogenetically, *R. belizensis* Nakasone, K. Draeger & B. Ortiz is closely related to *R. nakasoneae* but differs by having thicker basidiomes, encrusted hyphae, and distribution in Belize (Nakasone et al., 2017). *Rhizochaete fissurata* C.L. Zhao, recently described from China, formed a sister lineage to *R. nakasoneae* and *R. belizensis* but differs from *R. nakasoneae* in having thicker basidiomes with a cracked hymenophore, thinner cystidia walls (<1 μm), and slightly larger basidiospores (3–4.5 × 2.5–3 μm, Gu and Zhao, 2021). *Rhizochaete radicata* differs from *R. nakasoneae* by longer cystidia (60–100 μm) and slightly larger basidiospores (4–5 × 2.5–3 μm, Greslebin et al., 2004).

#### *Rhizochaete subradicata* Yue Li & S.H. He, sp. nov.

MycoBank: MB846342

Diagnosis—The species is recognized by a smooth hymenophore turning reddish brown in KOH, the presence of long lamprocystidia, and small ellipsoid basidiospores.

Type—China, Yunnan Province, Yongping City, Baotaishan Forest Park, on an angiosperm stump, 27 November 2015, He 3213 (BJFC 021608, holotype).

Etymology—Refer to the morphological similarity and close phylogenetic relationship of *R. radicata*.

Fruiting body—Basidiomes annual, resupinate, widely effused, loosely adnate, easily separated from substrate, and membranaceous to pellicular, first as small patches, later confluent up to 6 cm long, 3 cm wide, and up to 200 μm thick in section. Hymenophore smooth, light orange (5A5) to grayish orange (5B5), turning reddish brown in KOH, and not cracked upon drying; the margin thinning out, adnate, fimbriate, sterile, white or pale orange when juvenile, and slightly darkening with age; hyphal cords present, brownish yellow, and turning reddish brown in KOH; and context is pale yellow.

Microscopic structures—Hyphal system monomitic; generative hyphae simple-septate. Subiculum distinct, a loose texture; hyphae colorless, slightly thick-walled, smooth, frequently branched and septate, more or less parallel to substrate, and 3–6 μm in diameter. Subhymenium thickening, composed of lamprocystidia and hyphae; hyphae colorless, slightly thick-walled, smooth, more or less vertically arranged, moderately branched and septate, and 2.5–4 μm in diameter. Lamprocystidia numerous, subcylindrical to subfusiform, slightly thick-walled, encrusted with crystals in the upper part, mostly embedded or slightly projecting beyond the hymenium, and 40–75 (−90) × 7–15 μm (crystals included), with walls up to 1.5–2 μm. Basidia clavate, colorless, thin-walled, smooth, with a basal simple septum and four sterigmata, and 25–40 × 4–6 μm; basidioles in shape similar to basidia, but slightly smaller. Basidiospores ellipsoid, with an apiculus, colorless, thin-walled, smooth, usually with one or two oil drops, IKI–, CB–, (3.5–) 3.8–4.5 (−4.8) × 2.2–2.8 μm, L = 4.2 μm, W = 2.4 μm, and Q = 1.64–1.82 (*n* = 90/3).

Additional specimens examined—China, Guizhou Province, Jiangkou County, Fanjingshan Nature Reserve, on a rotten angiosperm trunk, 11 July 2018, He 5561 (BJFC 026622); Hubei Province, Wufeng County, Houhe Nature Reserve, on a fallen angiosperm trunk, 16 August 2017, He 5086 (BJFC 024604, CFMR); Hunan Province, Dong'an County, Shunhuangshan Nature Reserve, on a rotten angiosperm stump, 13 July 2015, He 2377 (BJFC 020831, CFMR); Jilin Province, Jiaohe County, forestry experimental area, on a fallen angiosperm trunk, 3 September 2017, He 5152 (BJFC 024670, CFMR); Jiangxi Province, Ji'an County, Jinggangshan Forest Park, on a fallen *Rhododendron* trunk, 11 August 2016, He 4282 (BJFC 023724, CFMR); Liaoning Province, Qingyuan County, Qingyuan forest ecological test station, on a dead angiosperm bark, 26 August 2015, He 2958 (BJFC 022025); Yunnan Province, Lushui County, Gaoligongshan Nature Reserve, on a fallen angiosperm trunk, 30 November 2015, He 3424 (BJFC 021820, CFMR); Yongde County, Daxueshan Nature Reserve, Dabaoshan, on a dead *Quercus* stump, 27 August 2015, He 2672 (BJFC 021111, CFMR); Yongping County, Baotaishan Forest Park, on a dead angiosperm tree, 27 November 2015, He 3246 (BJFC 021641).

**Figure 10:**
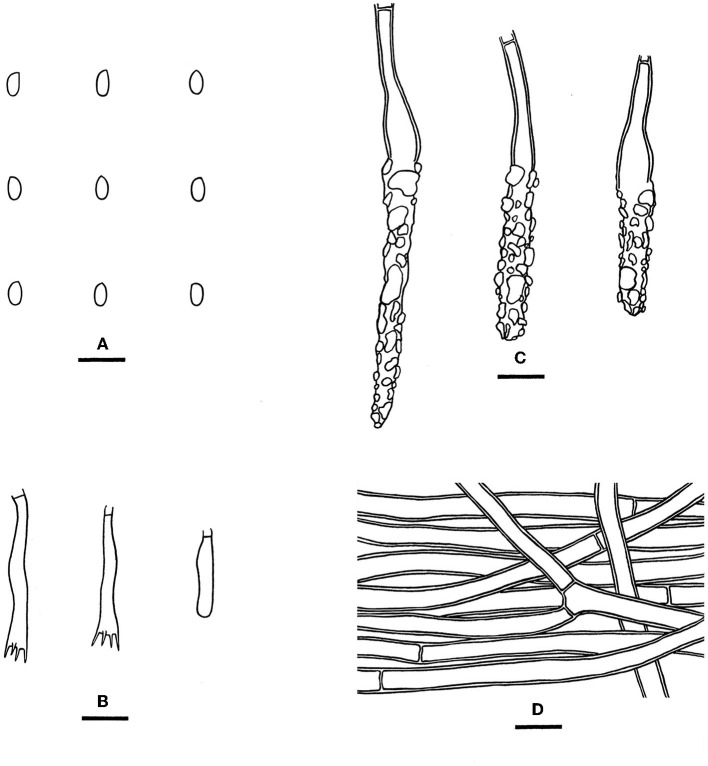
*Rhizochaete subradicata* [from the holotype He 3213; scale bars: **(A–D)** = 10 μm]. **(A)** Basidiospores; **(B)** Basidia and a basidiole; **(C)** Lamprocystidia; **(D)** Hyphae from subiculum.

Notes—*Rhizochaete subradicata* (Figures 8B, C, 10) is characterized by the thin pellicular basidiomes turning reddish brown in KOH, relatively long cystidia with thickened walls, and ellipsoid basidiospores. Morphologically and phylogenetically, *R. subradicata* is closely related to *R. radicata*, which differs by having thicker basidiomes and longer cystidia (60–100 μm, Nakasone et al., 1994). *Rhizochaete radicata* has been reported worldwide (Nakasone et al., 1994, 2017), but here we show that the widely distributed species in China is *R. subradicata*. Also, the occurrence of *R. radicata* in India, Japan, and Vietnam needs further study. *Rhizochaete grandinosa* C.L. Zhao & Z.R. Gu, recently described from Yunnan Province, southwestern China, differs from *R. subradicata* by having smaller cystidia (24–50 × 4–9 μm) and shorter basidia (14.5–21 μm, Gu and Zhao, 2021).

#### *Rhizochaete terrestris* Yue Li & S.H. He, sp. nov.

MycoBank: MB846343

Diagnosis—The species is recognized by a discontinuous hymenophore with well-developed hyphal cords, the absence of cystidia, and growth on the ground.

Type—China, Beijing, Haidian District, Yuyuantan Park, on the ground, 12 August 2022, He 7713 (BJFC 038849, holotype).

Etymology—Refer to the habitat on the ground.

Fruiting body—Basidiomes annual, resupinate, widely effused, adnate, on soil or rotten twigs and leaves, membranaceous or pellicular, fragile, the colonies up to 30 × 20 cm, up to 240 μm thick in section. Hymenophore discontinuous, smooth in the developed part, light yellow (4B5) to yellowish orange (4B7), turning pale purple in KOH, not cracked upon drying; the margin thinning out, adnate, fimbriate, and white; hyphal cords well developed, white to pale orange, and turning pale purple in KOH; and context is white.

Microscopic structures—Hyphal system monomitic; generative hyphae simple-septate with single clamps occasionally present. Subiculum distinct; hyphae colorless, thin- to slightly thick-walled, slightly encrusted with fine crystals, frequently branched and septate, more or less parallel to the substrate, and 2.5–7 μm in diameter; Subhymenium indistinct. Cystidia not observed. Basidia subclavate, colorless, thin-walled, smooth, with a basal simple septum and four sterigmata, and 28–38 × 4.5–6.5 μm; basidioles in shape similar to basidia, but slightly smaller. Basidiospores ellipsoid to broadly ellipsoid, with an apiculus, colorless, thin-walled, smooth, usually with two oil drops, IKI–, CB–, (3.8–) 4–5 (−5.2) × 2.5–3.2 (−3.5) μm, L = 4.4 μm, W = 2.9 μm, and Q = 1.47–1.56 (*n* = 60/2).

Additional specimens examined—China, Beijing, Haidian District, Jiufeng Forest Park, on the ground, 5 August 2020, He 6694 (BJFC 033642, holotype).

**Figure 11:**
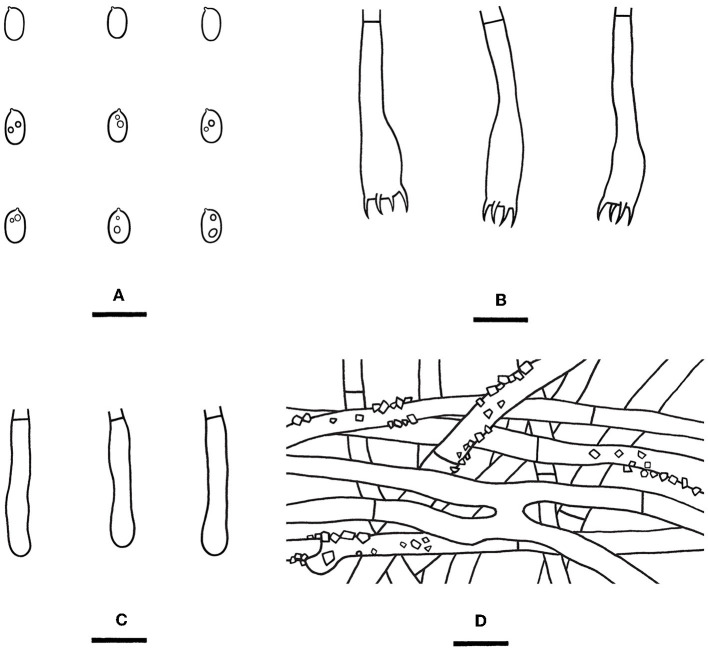
*Rhizochaete terrestris* [from the holotype He 7713; scale bars: **(A–D)** = 10 μm]. **(A)** Basidiospores; **(B)** Basidia; **(C)** Basidioles; **(D)** Hyphae from subiculum.

Notes—*Rhizochaete terrestris* (Figures 8D, E, 11) is characterized by the discontinuous basidiomes growing on the ground, well-developed hyphal cords, and the absence of cystidia. *Rhizochaete lutea* (Sheng H. Wu) C.C. Chen & Sheng H. Wu also lacks cystidia but differs from *R. terrestris* by the well-developed basidiomes unchanging in KOH, smaller basidia (19–23.5 × 3.7–4.2 μm), slightly narrower basidiospores (2–2.5 μm), and growth on bamboo in tropical areas (Wu, 1990). In the phylogenetic tree, *R. terrestris* formed a distinct lineage among the strongly supported group (93/83/1, Figure 3) that includes *R. radicata* and several other newly described species.

#### *Rhizochaete yunnanensis* Yue Li & S.H. He, sp. nov.

MycoBank: MB846344

Diagnosis—The species is recognized by a smooth hymenophore turning reddish brown in KOH and the presence of apically encrusted lamprocystidia, long basidia, and ellipsoid basidiospores.

Type—China, Yunnan Province, Lushui County, Gaoligongshan Nature Reserve, on a fallen angiosperm trunk, 28 November 2015, He 3302 (BJFC 021697, holotype; CFMR, isotype).

Etymology—Refer to the type locality in Yunnan Province, southwestern China.

Fruiting body—Basidiomes annual, resupinate, widely effused, loosely adnate, easily separated from the substrate, and membranaceous to pellicular, first as small patches, later confluent up to 5 cm long, 2 cm wide, and up to 160 μm thick in section. Hymenophore smooth, grayish yellow (4B4) to brownish orange (5B6), turning reddish brown in KOH, and not cracked upon drying; the margin thinning out, adnate, fimbriate, paler than hymenophore surface, and cream; hyphal cords yellow and turning reddish brown in KOH; and context is yellow.

Microscopic structures—Hyphal system monomitic; generative hyphae simple-septate with rare single clamps. Subiculum distinct; hyphae colorless, thin- to slightly thick-walled, smooth, frequently branched, sometimes with H- or Y-connections, frequently septate, loosely interwoven, and 3.5–6 μm in diameter. Subhymenium thickening, composed of lamprocystidia and hyphae, hyphae colorless, thin- to slightly thick-walled, smooth, loosely interwoven, moderately branched, rarely septate, and 1.5–3 μm in diameter. Lamprocystidia is numerous, subfusiform with an obtuse apex, apically encrusted, mostly embedded, and 40–52 × 6–10 μm (crystals included), with walls up to 1–1.5 μm. Basidia clavate, colorless, thin-walled, smooth, with a basal simple septum and four sterigmata, and 33–45 × 4.5–7 μm; basidioles in shape similar to basidia, but slightly smaller. Basidiospores ellipsoid, with an apiculus, colorless, thin-walled, smooth, usually with oil drops, IKI–, CB–, 4.2–5.2 × 2.8–3.2 μm, L = 4.8 μm, W = 3 μm, and Q = 1.6 (*n* = 30/1).

**Figure 12:**
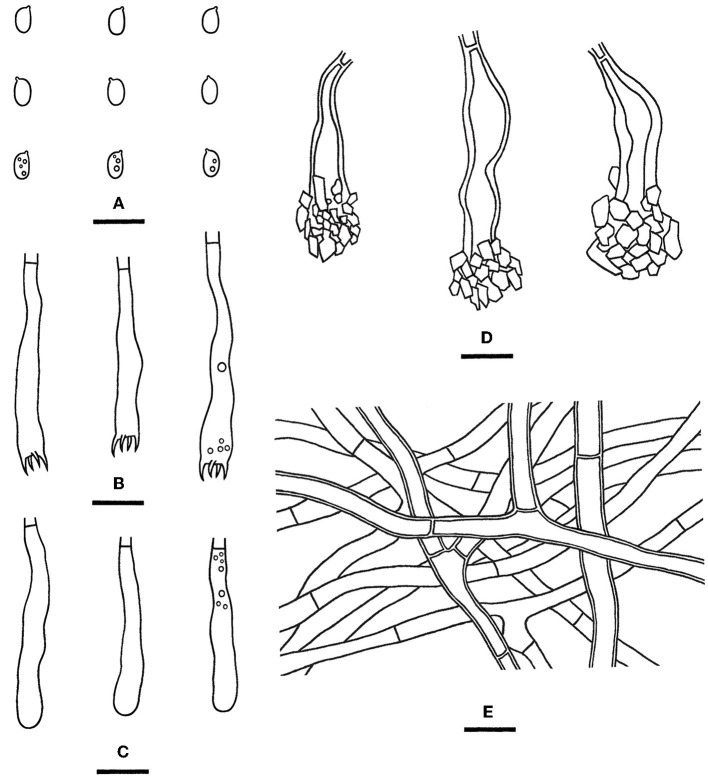
*Rhizochaete yunnanensis* [from the holotype He 3302; scale bars: **(A–E)** = 10 μm]. **(A)** Basidiospores; **(B)** Basidia; **(C)** Basidioles; **(D)** Lamprocystidia; **(E)** Hyphae from subiculum.

Notes—*Rhizochaete yunnanensis* (Figures 8F, 12) is characterized by thin basidiomes turning reddish brown in KOH, apically encrusted thick-walled cystidia, and long basidia. In the phylogenetic tree (Figure 3), *R. yunnanensis* is the sister to *R. violascens* (Fr.) K.H. Larss., which differs in having a violaceous hymenophore and clamped generative hyphae and lacking cystidia (Eriksson and Ryvarden, 1973). *Rhizochaete subradicata* is similar to *R. yunnanensis* by sharing similar-sized basidia and basidiospores but differs in having larger cystidia (40–75 × 7–15 μm) with more proportions of the length encrusted.

#### *Phanerochaete subsanguinea* Yue Li & S.H. He, nom. nov.

MycoBank: MB846345

Replaced synonym—*Phanerochaete rhizomorpha* C.L. Zhao & D.Q. Wang, Journal of Fungi 7: 10. 2021. [MB#841272]

Etymology—Refer to the morphological similarity and close phylogenetic relationship of *P. sanguinea* (Fr.) Pouzar.

Description—See Wang and Zhao (2021).

**Figure 13:**
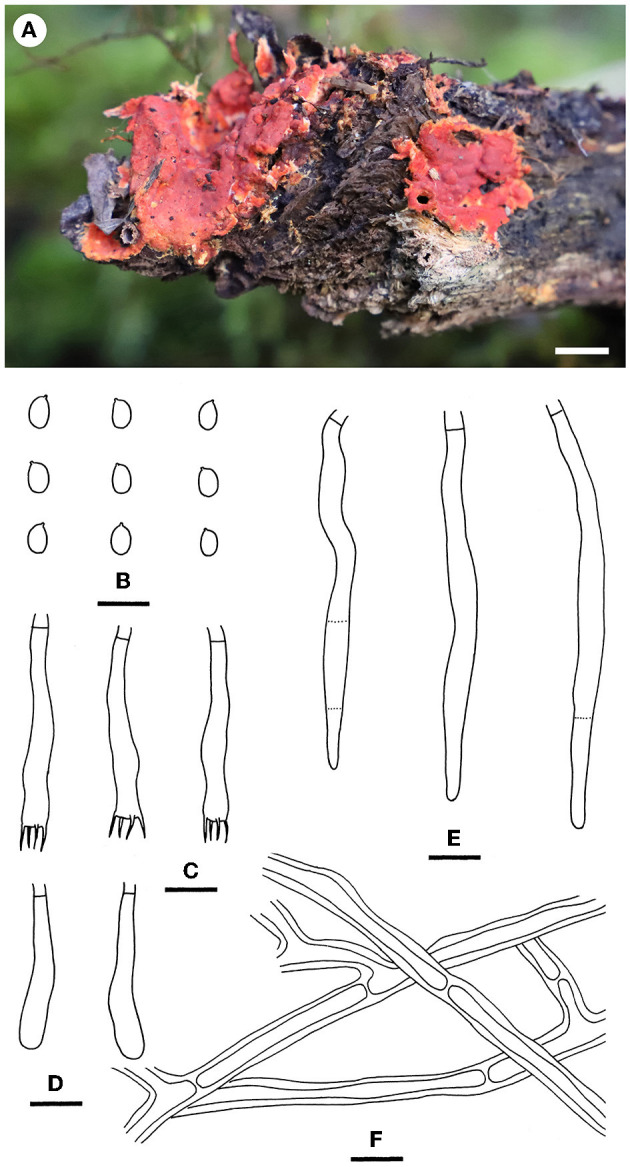
*Phanerochaete subsanguinea* [from He 6131; scale bars: **(A)** = 1 cm; **(B–F)** = 10 μm]. **(A)** Basidiomes; **(B)** Basidiospores; **(C)** Basidia; **(D)** Basidioles; **(E)** Cystidia; **(F)** Hyphae from subiculum.

Notes—According to Art. 53.1, the name *P. rhizomorpha* C.L. Zhao & D.Q. Wang published on 11 December 2021 is an invalid name since it is a homonym of *P. rhizomorpha* C.C. Chen, Sheng H. Wu & S.H. He published on 15 November 2021 (Chen et al., 2021; Wang and Zhao, 2021). Our morphological and phylogenetic studies on the representative specimens showed that the two names represented two distinct species (Figure 4). Therefore, we propose the new name *P. subsanguinea* (Figure 13) to replace *P. rhizomorpha* C.L. Zhao & D.Q. Wang.

## Discussion

The phylogeny of the Phanerochaetaceae at the generic level is becoming much clearer with some new genera introduced for the independent lineages (Floudas and Hibbett, 2015; Miettinen et al., 2016; Yuan et al., 2017; Chen et al., 2018b, 2021; Ma and Zhao, 2019). At present, the family includes 23 genera, most of which are corticioid fungi (Chen et al., 2021). Although most of the newly described taxa in the family originated from East Asia, some groups in this area still need more systematic studies based on more complete samplings.

Chen et al. (2021) used *Roseograndinia* for a distinct lineage of two new species, which are morphologically similar to the type, *R. rosea* (Henn.) Hjortstam & Ryvarden. Morphologically, the genus is similar to *Hyphodermella*, which differs by having encrusted cystidioid hyphal ends. Our phylogenetic analyses based on more samples of the two genera showed that two recently described species without cystidioid hyphal ends, *Hyphodermella aurantica* and *H. zixishanensis*, nested within the *Roseograndinia*. Meanwhile, two new species, *H. laevigata* and *H. tropica*, in the core clade of *Hyphodermella* were found, though the former species also lacks cystidioid hyphal ends. The poroid species, *Hyphodermella poroides* Y.C. Dai & C.L. Zhao, did not nest within the core group of the genus but clustered with *Geliporus exilisporus* (Y.C. Dai & Niemelä) Yuan, Jia J. Chen & S.H. He. However, their relationship was not well supported, and there are clear morphological differences between the two species (Yuan et al., 2017; Zhao et al., 2017). At present, we accept a broad concept of *Hyphodermella* to include nine species as follows:

**A key to accepted species of** ***Hyphodermella*** **s.l**.

1. Hymenophore poroid ………………………..*H. poroides*

1. Hymenophore non-poroid ……………………………2

2. Hymenophore smooth to tuberculate ……………………3

2. Hymenophore grandinioid, odontoid to hydnaceous ……….4

3. Cystidioid hyphal ends absent, reported in southern China …………………………………………...*H. laevigata*

3. Cystidioid hyphal ends present, reported in Far East of Russia ……………………………………..*H. pallidostraminea*

4. Context distinctly brown ………………*H. brunneocontexta*

4. Context cream, pale yellow ……………………………5

5. Basidiospores <6 μm long …………………….*H. tropica*

5. Basidiospores > 6 μm long ……………………………6

6. Basidiospores narrowly ellipsoid, <4 μm wide ………………………………………*H. maunakeaensis*

6. Basidiospores ellipsoid, > 4 μm wide ……………………7

7. Basidiomes up to 500 μm thick, basidia up to 60 μm long …………………………………………..*H. corrugata*

7. Basidiomes up to 110 μm thick, basidia up to 40 μm long ……………………………………………………8

8. Subicular hyphae agglutinated, widely distributed in Mediterranean area and China on many hosts ………………………………………………*H. rosae*

8. Subicular hyphae separable, found in Italy on ………………………………….*Ampelopsis H. ochracea*

*Rhizochaete* is morphologically well-circumscribed and phylogenetically well-supported in the Phanerochaetaceae. It contains 17 species worldwide, including four new species recently described from China (Nakasone et al., 2017; Chen et al., 2021; Gu and Zhao, 2021). Our analyses herein demonstrated that the species diversity of the genus in China is rich, and four additional new species were found based mainly on molecular evidence. Meanwhile, according to our investigation records, some species are abundantly distributed locally, for example, *R. chinensis* C.C. Chen, Sheng H. Wu & S.H. He is one of the most common corticioid species in the Beijing area. The species, *R. sulphurina* (P. Karst.) K.H. Larss., was recorded in northeastern China earlier [as *Ceraceomyces sulphurinus* (P. Karst.) J. Erikss. & Ryvarden, Dai, 2011], but we did not recover this species based on our investigations and analyses. Thus, we accepted eight species of *Rhizochaete* in China as follows:

**A key to species of** ***Rhizochaete*** **in China**

1. Cystidia absent ……………………………………..2

1. Cystidia present …………………………………….3

2. Basidiomes on wood or bamboo, color unchanged in KOH, found in tropical areas ……………………………*R. lutea*

2. Basidiomes on the ground, turning purple in KOH, found in temperate area ……………………………….*R. terrestris*

3. Basidiospores broadly ellipsoid to ovoid, usually <2.5 μm wide ………………………………………....*R. nakasoneae*

3. Basidiospores ellipsoid, usually > 2.5 μm wide …………...4

4. Basidiomes turning purple in KOH …………………….5

4. Basidiomes turning reddish brown in KOH ………………6

5. Cystidia 18–60.5 × 6–11 μm, basidia 11–33 × 3–5 μm ……………………………………………*R. fissurata*

5. Cystidia 20–50 × 4–9 μm, basidia 14.5–21 × 4.3–5.2 μm ………………………………………….*R. grandinosa*

6. Basidiomes up to 500 μm thick, basidia <27 μm long ……………………………………………*R. chinensis*

6. Basidiomes up to 200 μm thick, basidia mostly > 27 μm long ……………………………………………………7

7. Cystidia 40–75 × 7–15 μm, basidiospores 3.8–4.5 × 2.2–2.8 μm …………………………………………*R. subradicata*

7. Cystidia 40–52 × 6–10 μm, basidiospores 4.2–5.2 × 2.8–3.2 μm …………………………………………*R. yunnanensis*

## Data availability statement

The data presented in the study are deposited in the NCBI and MycoBank repositories, accession numbers ON963990–ON964026, OP549029, OP549030, OP102693, OP102695 for NCBI repository, and accession numbers MB846336–MB846345 for MycoBank repository.

## Author contributions

YL performed the phylogenetic analyses and did most of the measurements, descriptions, and illustrations. C-CC provided some specimens and sequences and revised the manuscript. S-HH designed the research, collected most of the specimens, and wrote and revised the manuscript. All authors contributed to the article and approved the submitted version.

## Footnotes

^1^ http://sweetgum.nybg.org/science/ih/

^2^ https://sites.duke.edu/vilgalyslab/rdna_primers_for_fungi/

^3^ https://www.ncbi.nlm.nih.gov/

^4^ http://mafft.cbrc.jp/alignment/server/

## References

[B1] Bernicchia A. Gorjón S. P. (2010). Corticiaceae s.l. Fungi Europaei 12. Edizioni Candusso. Alassio, Italia

[B2] Binder M. Larsson K. H. Matheny P. B. Hibbett D. S. (2010). Amylocorticiales ord. nov. and Jaapiales ord. nov.: early diverging clades of Agaricomycetidae dominated by corticioid forms. Mycologia 102, 865–880. 10.3852/09-288 20648753

[B3] Boonmee S. Wanasinghe D. N. Calabon M. S. Huanraluek N. Chandrasiri S. K. U. Jones G. E. B.. (2021). Fungal diversity notes 1387–1511: taxonomic and phylogenetic contributions on genera and species of fungal taxa. Fungal Div. 111, 1–335. 10.1007/s13225-021-00489-3 34899100 PMC8648402

[B4] Chen C. C. Chen C. Y. Lim Y. W. Wu S. H. (2020). Phylogeny and taxonomy of *Ceriporia* and other related taxa and description of three new species. Mycologia 112, 64–82. 10.1080/00275514.2019.1664097 31906813

[B5] Chen C. C. Chen C. Y. Wu S. H. (2021). Species diversity, taxonomy and multi-gene phylogeny of phlebioid clade (Phanerochaetaceae, Irpicaceae, Meruliaceae) of Polyporales. Fungal Div. 111, 337–442. 10.1007/s13225-021-00490-w

[B6] Chen C. C. Wu S. H. Chen C. Y. (2018a). Four species of polyporoid fungi newly recorded from Taiwan. Mycotaxon 133, 45–54. 10.5248/133.45

[B7] Chen C. C. Wu S. H. Chen C. Y. (2018b). *Hydnophanerochaete* and *Odontoefibula*, two new genera of phanerochaetoid fungi (Polyporales, Basidiomycota) from East Asia. MycoKeys 39, 75–96. 10.3897/mycokeys.39.28010 30271259 PMC6160836

[B8] Chen J. J. Wang Y. R. Wang C. G. Dai Y. C. (2022). Two new species of *Ceriporia* (Irpicaceae, Basidiomycota) from the Asia Pacific area. Mycological Progress 21, 39–48. 10.1007/s11557-021-01731-7

[B9] Chikowski R. S. Larsson K. H. Gibertoni T. B. (2016). Three new combinations in *Rhizochaete* (Agaricomycetes, Fungi) and a new record to the Brazilian Amazonia. Nova Hedwigia 102, 185–196. 10.1127/nova_hedwigia/2015/0298

[B10] Crous P. W. Osieck E. R. Jurjevic Ž. Boers J. van Iperen A. L. Starink-Willemse M.. (2021). Fungal Planet description sheets: 1284–1382. Persoonia 47, 178–374. 10.3767/persoonia.2021.47.06 PMC10486635 37693795

[B11] Dai Y. C. (2011). A revised checklist of corticioid and hydnoid fungi in China for 2010. Mycoscience 52, 69–79. 10.1007/S10267-010-0068-1

[B12] Darriba D. Taboada G. L. Doallo R. Posada D. (2012). jModelTest 2: more models, new heuristics and parallel computing. Nat. Methods 9, 772. 10.1038/nmeth.2109 22847109 PMC4594756

[B13] Duhem B. Buyck B. (2011). Hyphodermella brunneocontexta sp. nov. de l'île de Mayotte (France) (Basidiomycota, Polyporales). Cryptogamie Mycologie 32, 413–420. 10.7872/crym.v32.iss4.2011.413

[B14] Eriksson J. Ryvarden L. (1973). The Corticiaceae of North Europe. Cryptogamie Mycologie 2, 60–287.

[B15] Floudas D. Hibbett D. S. (2015). Revisiting the taxonomy of *Phanerochaete* (Polyporales, Basidiomycota) using a four gene dataset and extensive ITS sampling. Fungal Biology 119, 679–719. 10.1016/j.funbio.2015.04.003 26228559

[B16] Gilbertson R. L. (2001). Fungi from the mamane-naio vergetation zone of Hawaii. Fungal Div. 6, 35–68.

[B17] Greslebin A. Nakasone K. K. Rajchenberg M. (2004). *Rhizochaete*, a new genus of phanerochaetoid fungi. Mycologia 96, 260–271. 10.1080/15572536.2005.11832976 21148853

[B18] Gu Z. R. Zhao C. L. (2021). The hidden wood-decaying fungal diversity: *Rhizochaete* from East Asia. J. Fungi 13, 503. 10.3390/d13100503

[B19] Hall T. A. (1999). Bioedit: a user-friendly biological sequence alignment editor and analysis program for Windows 95/98/NT. Nucleic Acids Sympos. Series 41, 95–98.

[B20] Huang R. X. Zhao C. L. (2020). Three new species of *Phlebia* (Polyporales, Basidiomycota) based on the evidence from morphology and DNA sequence data. Mycologic. Progr. 19, 753–767. 10.1007/s11557-020-01591-7

[B21] Justo A. Miettinen O. Floudas D. Ortiz-Santana B. Sjökvist E. Lindner D.. (2017). A revised family-level classification of the Polyporales (Basidiomycota). Fung. Biol. 121, 798–824. 10.1016/j.funbio.2017.05.010 28800851

[B22] Katoh K. Rozewicki J. Yamada K. D. (2017). MAFFT online service: multiple sequence alignment, interactive sequence choice and visualization. Briefings in Bioinformatics 20, 1160–1166. 10.1093/bib/bbx108 28968734 PMC6781576

[B23] Kearse M. Moir R. Wilson A. Stones-Havas S. Cheung M. Sturrock S.. (2012). Geneious basic: an integrated and extendable desktop software platform for the organization and analysis of sequence data. Bioinformatics 28, 1647–1649. 10.1093/bioinformatics/bts199 22543367 PMC3371832

[B24] Kornerup A. Wanscher J. H. (1978). Methuen handbook of colour. London: E. Methuen and Co., Ltd.

[B25] Larsson K. H. (2007). Re-thinking the classifcation of corticioid fungi. Mycolog. Res. 111, 1040–1063. 10.1016/j.mycres.2007.08.001 17981020

[B26] Li T. Gao J. L. Huang J. H. Gu L. Zou J. Wu X. J. (2021). Phlebiopsis xuefengensis sp. nov. from Gastrodia elata (Orchidaceae) in Hunan Province, Southern China. South Afric. J. Bot. 142, 299–304. 10.1016/j.sajb.2021.06.034

[B27] Li Y. He S. H. Chen C. C. Nakasone K. K. Ma H. X. (2022). Global taxonomy and phylogeny of Irpicaceae (Polyporales, Basidiomycota) with descriptions of seven new species and proposals of two new combinations. Front. Microbiol. 13, 1–26. 10.3389/fmicb.2022.911978 35794917 PMC9251475

[B28] Lim Y. W. Jung H. S. (2003). Irpex hydnoides, sp. *nov*. is new to science, based on morphological, cultural and molecular characters. Mycologia 95, 694–699. 10.1080/15572536.2004.11833073 21148978

[B29] Lima V. X. Sousa L. C. dos Santos C. R. Santos C. Lima N. Gibertoni T. B. (2020). Additions to neotropical stereoid fungi (Polyporales, Basidiomycota): one new species of *Lopharia* and one new combination in *Phlebiopsis*. Mycologic. Progr. 19, 31–40. 10.1007/s11557-019-01538-7

[B30] Lira C. R. S. Chikowski R. S. de Lima V. X. Gibertoni T. B. Larsson K. H. (2022). *Allophlebia*, a new genus to accomodate *Phlebia ludoviciana* (Agaricomycetes, Polyporales). Mycologic. Progr. 21, 1–11. 10.1007/s11557-022-01781-5

[B31] Ma X. Zhao C. L. (2019). Crepatura ellipsospora gen. *et sp. nov. in* Phanerochaetaceae (Polyporales, Basidiomycota) bearing a tuberculate hymenial surface. Mycologic. Progr. 18, 785–793. 10.1007/s11557-019-01488-0

[B32] Maddison W. P. Maddison D. R. (2018). Mesquite: a modular system for evolutionary analysis. Version 3.5.1. Available online at: http://www.mesquiteproject.org (accessed October 01, 2022).

[B33] Miettinen O. Spirin V. Vlasák J. Rivoire B. Stenroos S. Hibbett D. (2016). Polypores and genus concepts in Phanerochaetaceae (Polyporales, Basidiomycota). MycoKeys 17, 1–46. 10.3897/mycokeys.17.10153

[B34] Nakasone K. K. Bergman C. R. Burdsall H. H. (1994). *Phanerochaete filamentosa—Corticium radicatum* species complex in North America. Sydowia 46, 44–62.

[B35] Nakasone K. K. Draeger K. R. Ortiz-Santana B. (2017). A contribution to the taxonomy of *Rhizochaete* (Polyporales, Basidiomycota). Cryptogam Mycologie 38, 81–99. 10.7872/crym/v38.iss1.2017.81

[B36] Nakasone K. K. Ortiz-Santana B. He S. H. (2021). Taxonomic studies of crust fungi with spines in *Radulomyces, Sarcodontia*, and the new genus *Noblesia*. Mycologic. Progr. 20, 1479–1501. 10.1007/s11557-021-01746-0

[B37] Parmasto E. Hallenberg N. (2000). A taxonomic study of phlebioid fungi (Basidiomycota). Nordic J. Bot. 20, 105–118. 10.1111/j.1756-1051.2000.tb00740.x

[B38] Phookamsak R. Hyde K. D. Jeewon R. Bhat D. J. Gareth Jones E. B. Maharachchikumbura S. S. N.. (2019). Fungal diversity notes 929–1035: taxonomic and phylogenetic contributions on genera and species of fungi. Fung. Diver. 95, 1–273. 10.1007/s13225-019-00421-w

[B39] Ronquist F. Teslenko M. van der Mark P. Ayres D. L. Darling A. Hohna S.. (2012). MrBayes 3.2: efficient Bayesian phylogenetic inference and model choice across a large model space. Systematic Biol. 61, 539–542. 10.1093/sysbio/sys029 22357727 PMC3329765

[B40] Spirin V. Volobuev S. Okun M. Miettinen O. Larsson K. H. (2017). What is the type species of *Phanerochaete* (Polyporales, Basidiomycota). Mycologic. Progr. 16, 171–183. 10.1007/s11557-016-1267-8

[B41] Stamatakis A. (2014). RAxML Version 8: A tool for phylogenetic analysis and post-analysis of large phylogenies. Bioinformatics 30, 1312–1313. 10.1093/bioinformatics/btu033 24451623 PMC3998144

[B42] Swofford D. L. (2002). PAUP^*^*: Phylogenetic analysis using parsimony (*^*^*and other methods). Version 4.0b10. Sinauer Associates, Sunderland, Massachusetts*.

[B43] Telleria M. T. Dueñas M. Melo I. Martín M. P. (2010). Morphological and molecular studies of *Hyphodermella* in the Western Mediterranean area. Mycologic. Progr. 9, 585–596. 10.1007/s11557-010-0666-5

[B44] Wang D. Q. Zhao C. L. (2021). Morphological and phylogenetic evidence for recognition of two new species of *Phanerochaete* from East Asia. J. Fungi 7, 1063. 10.3390/jof7121063 34947045 PMC8706112

[B45] Wang H. Gu Z. Zhao C. L. (2021). *Hyphodermella zixishanensis* (Polyporales, Basidiomycota), a new species with reddish hymenial surface. Nordic J. Bot. 39, e03329. 10.1111/njb.03329

[B46] Wang H. Zhao C. L. (2020). Hyphodermella aurantiaca sp. *nova (*Polyporales, Basidiomycota) as evidenced by morphological characters and phylogenetic analyses. Annales Botanici Fennici 58, 61–68. 10.5735/085.058.0110

[B47] White T. J. Bruns T. Lee S. Taylor J. (1990). Amplification and direct sequencing of fungal ribosomal RNA genes for phylogenetics, in PCR Protocols: A Guide to Methods and Applications, eds Innis M.A. Gelfand D.H. Sninsky J.J. White T.J. (San Diego: Academic Press), 315–322.

[B48] Wu S. H. (1990). The Corticiaceae (Basidiomycetes) subfamilies Phlebioideae, Phanerochaetoideae and Hyphodermoideae in Taiwan. Acta Botanica Fennica 142, 1–123.

[B49] Wu S. H. Chen Y. P. Wei C. L. Floudas D. Dai Y. C. (2018a). Two new species of *Phanerochaete* (Basidiomycota) and redescription of P. robusta. Mycologic. Progr. 17, 425–435. 10.1007/s11557-017-1368-z

[B50] Wu S. H. Nilsson H. R. Chen C. T. Yu S. Y. Hallenberg N. (2010). The white-rotting genus *Phanerochaete* is polyphyletic and distributed throughout the phleboid clade of the Polyporales (Basidiomycota). Fungal Diver. 42, 107–118. 10.1007/s13225-010-0031-7

[B51] Wu S. H. Wang D. M. Chen Y. P. (2018b). *Purpureocorticium microsporum* (Basidiomycota) gen. et sp. nov. from East Asia. Mycologic. Progr. 17, 357–364. 10.1007/s11557-017-1362-536059897

[B52] Xu T. M. Zeng Y. F. Cheng Y. H. Zhao C. L. (2020). *Phlebiopsis lacerata* sp. *nov. (Polyporales, Basidiomycota)* from southern China. Phytotaxa 440, 268–280. 10.11646/phytotaxa.440.4.2

[B53] Xu Y. L. Cao Y. F. Nakasone K. K. Chen C. C. He S. H. (2020). Taxonomy and phylogeny of *Phanerochaete* sensu stricto (Polyporales, Basidiomycota) with emphasis on Chinese collections and descriptions of nine new species. Mycosphere 11, 1527–1552. 10.5943/mycosphere/11/1/12

[B54] Yuan Y. Chen J. J. He S. H. (2017). Geliporus exilisporus gen. *et comb. nov.*, a xanthochroic polypore in Phanerochaetaceae from China. Mycoscience 58, 197–203. 10.1016/j.myc.2017.01.006

[B55] Zhao C. L. Liu X. F. Ma X. (2019). Phlebiopsis yunnanensis sp. *nov. (Polyporales, Basidiomycota)* evidenced by morphological characters and phylogenetic analysis. Nova Hedwigia 108, 265–279. 10.1127/nova_hedwigia/2018/0508

[B56] Zhao C. L. Ren G. J. Wu F. (2017). A new species of *Hyphodermella* (Polyporales, Basidiomycota) with a poroid hymenophore. Mycoscience 58, 452–456. 10.1016/j.myc.2017.06.007

[B57] Zhao Y. N. He S. H. Nakasone K. K. Wasantha Kumara K. L. Chen C. C. Liu S. L.. (2021). Global phylogeny and taxonomy of the wood-decaying fungal genus *Phlebiopsis* (Polyporales, Basidiomycota). Front. Microbiol. 12, 622460. 10.3389/fmicb.2021.622460 33643251 PMC7902713

